# Systematic analysis of the Rboh gene family in seven gramineous plants and its roles in response to arbuscular mycorrhizal fungi in maize

**DOI:** 10.1186/s12870-023-04571-7

**Published:** 2023-11-30

**Authors:** Fulang Wu, Manli Zhao, Yajing Zhang, Weina Si, Beijiu Cheng, Xiaoyu Li

**Affiliations:** 1 National Engineering Laboratory of Crop Stress Resistance Breeding, Anhui Agricultural University, Hefei, 230036 China; 2 College of Life Science, Anhui Agricultural University of China, Changjiang West Road, Hefei, 230036 China

**Keywords:** Rboh gene, Arbuscular mycorrhiza fungi (AMF), Symbiosis, Gramineous

## Abstract

**Background:**

Plant respiratory burst oxidase homolog (*Rboh*) gene family produces reactive oxygen species (ROS), and it plays key roles in plant–microbe interaction. Most *Rboh* gene family-related studies mainly focused on dicotyledonous plants; however, little is known about the roles of *Rboh* genes in gramineae*.*

**Results:**

A total of 106 *Rboh* genes were identified in seven gramineae species, including *Zea mays*, *Sorghum bicolor*, *Brachypodium distachyon*, *Oryza sativa*, *Setaria italica*, *Hordeum vulgare*, and *Triticum aestivum*. The Rboh protein sequences showed high similarities, suggesting that they may have conserved functions across different species. Duplication mode analysis detected whole-genome/segmental duplication (WGD)/(SD) and dispersed in the seven species. Interestingly, two local duplication (LD, including tandem and proximal duplication) modes were found in *Z. mays*, *S. italica and H. vulgare*, while four LD were detected in *T. aestivum*, indicating that these genes may have similar functions. Collinearity analysis indicated that *Rboh* genes are at a stable evolution state in all the seven species. Besides, *Rboh* genes from *Z. mays* were closely related to those from *S. bicolor*, consistent with the current understanding of plant evolutionary history. Phylogenetic analysis showed that the genes in the subgroups I and II may participate in plant-AM fungus symbiosis. Cis-element analysis showed that different numbers of elements are related to fungal induction in the promoter region. Expression profiles of *Rboh* genes in *Z. mays* suggested that *Rboh* genes had distinct spatial expression patterns. By inoculation with AM fungi, our transcriptome analysis showed that the expression of *Rboh* genes varies upon AM fungal inoculation. In particularly, *ZmRbohF* was significantly upregulated after inoculation with AM fungi. p*ZmRbohF*::GUS expression analyses indicated that *ZmRbohF* was induced by arbuscular mycorrhizal fungi in maize. By comparing WT and *ZmRbohF* mutant, we found *ZmRbohF* had limited impact on the establishment of maize-AM fungi symbiosis, but play critical roles in regulating the proper development of arbuscules.

**Conclusions:**

This study provides a comprehensive analysis of the evolution relationship of *Rboh* genes in seven gramineae species. Results showed that several *Rboh* genes regulate maize-AM fungal symbiosis process. This study provides valuable information for further studies of *Rboh* genes in gramineae.

**Supplementary Information:**

The online version contains supplementary material available at 10.1186/s12870-023-04571-7.

## Background

Plant NADPH oxidases, also known as respiratory burst oxidase homolog (Rboh), encode a homolog of phagocyte gp91phox [1]. Rbohs are located in the plasma membrane and they contain six transmembrane domains with two haem groups, C-terminal FAD-binding domain, NADPH hydrophilic domains, and two N-terminal calcium-binding EF-hand motifs. The extended N-terminal region of Rbohs in plants contains two putative calcium-binding domains (EF-hands), which are directly regulated by Ca^2+^ [2]. Following this initial discovery, Rboh genes have been identified in many plant species, including *Arabidopsis thaliana* [3], *Medicago truncatula* [4], tomato [5], and potato [6]. In plants, Rboh is activated by C-terminal or N-terminal phosphorylation [7], to catalyze the production of ROS in response to external diverse stimuli, and is one of the primary regulators of ROS production [8]. It has been reported that Rboh genes play a role in plant development, including cell amplification, seed germination, pollen tube elongation, and lateral root development [9–11]. Rboh genes also play a role in responses to abiotic and biotic stress. For instance, *NtbHLH123* activates *NtRbohE* by binding to the E-box motif in the promoter region of *NtRbohE*, improving salt tolerance in tobacco [12]. The early production of H_2_O_2_ by *AtRbohD* and *AtRbohF* is required for salinity-induced antioxidant defense responses [13]. In addition, *RbohA* plays critical roles in Rhizobium infection and nodule maturation in Common Bean [14]. CDPK-Rboh complex enhances rhizobial colonization in Medicago truncatula nodules by suppressing the innate immunity [15]. The function of *AtRbohD* and *AtRbohF* involve the accumulation of reactive oxygen intermediates during the plant defense response [16]. *NtRbohD*, *NbRbohA*, and *NbRbohB* participate in ROS accumulation and resistance to fungal pathogens [17].

Arbuscular mycorrhizal (AM) symbiont is one of the most ancient and widespread symbioses in nature [18, 19]. Arbuscular mycorrhizal fungi (AMF) are obligate trophical fungi and some of the most abundant organisms on earth. AMF form symbiotic associations with most plants and are found in almost all vegetation systems from the sub-polar regions to the tropical rain forests, and even in some aquatic ecosystems [20, 21]. The establishment of AM symbionts depends on the precise "molecular communication" between the host plant and AM fungi. The main advantage of AM symbiosis is the exchange of nutrients [22, 23]. In addition, plants colonized by AMF often show higher tolerance to biotic and abiotic stresses compared with nonmycorrhizal plants and this is not a mere consequence of a better nutritional status [23, 24]. Given the global environmental changes, researches focus have gradually shifted to AM-plant symbiosis to provide an important reference for studying Rboh genes. Recently, Rboh has been reported to affect the colonization and development of AM fungi in legumes, including *M. truncatula*. *MtRbohE* mainly mediates the development of arbuscules within the host root cells [25]. In *P. vulgaris*, *PvRbohB* negatively regulates mycorrhizal colonization [26]. However, studies on Rboh in other species remain scanty.

Gramineae crops have high economic and numerous nutritional values, and they are widely used in scientific research because of their high genetic diversity and genomic data [27]. *Zea mays* (maize or corn) is the most important crop in the world, and it has stronger environmental adaptability and higher yield compared with traditional food crops. Maize is an ideal model for studying higher plants and animals, including the evolutionary processes, domestication, development, and cell destinies [28]. To gain a comprehensive deeper understanding of the *Rboh* gene family, genome-wide analysis of the *Rboh* gene family was performed by sequencing and annotating the genomes for *Z. mays*, *S. bicolor*, *B. distachyon*, *O. sativa*, *S. italica*, *H. vulgare*, and *T. aestivum*. Basic information about the evolution and expression patterns of Rbohs was revealed. Members of the Rboh gene family in *P. vulgaris* [26], *M. truncatula* [29], *A. thaliana* [16], and *N. Benthamiana* [17] are involved in establishing symbiotic associations and activating immune defenses. The effect of AM on the expression of *ZmRboh* genes was analyzed using quantitative real-time PCR (qRT-PCR) and sequencing. The expression of p*ZmRbohF*::GUS was observed in the epidermis cells and endothelial cells near the vascular column of roots inoculated with AM fungi under low Pi conditions, *ZmRbohF* mutation affected the ratio of mature and small arbuscules in mycorrhizal and it plays an important role in arbuscular development in maize. Our results provide a reference for further functional analysis of the Rboh genes in gramineous plants, especially in AM symbiosis relationships.

## Results

### Identification of *Rboh* genes in seven gramineous plants

**Fig. 1:**
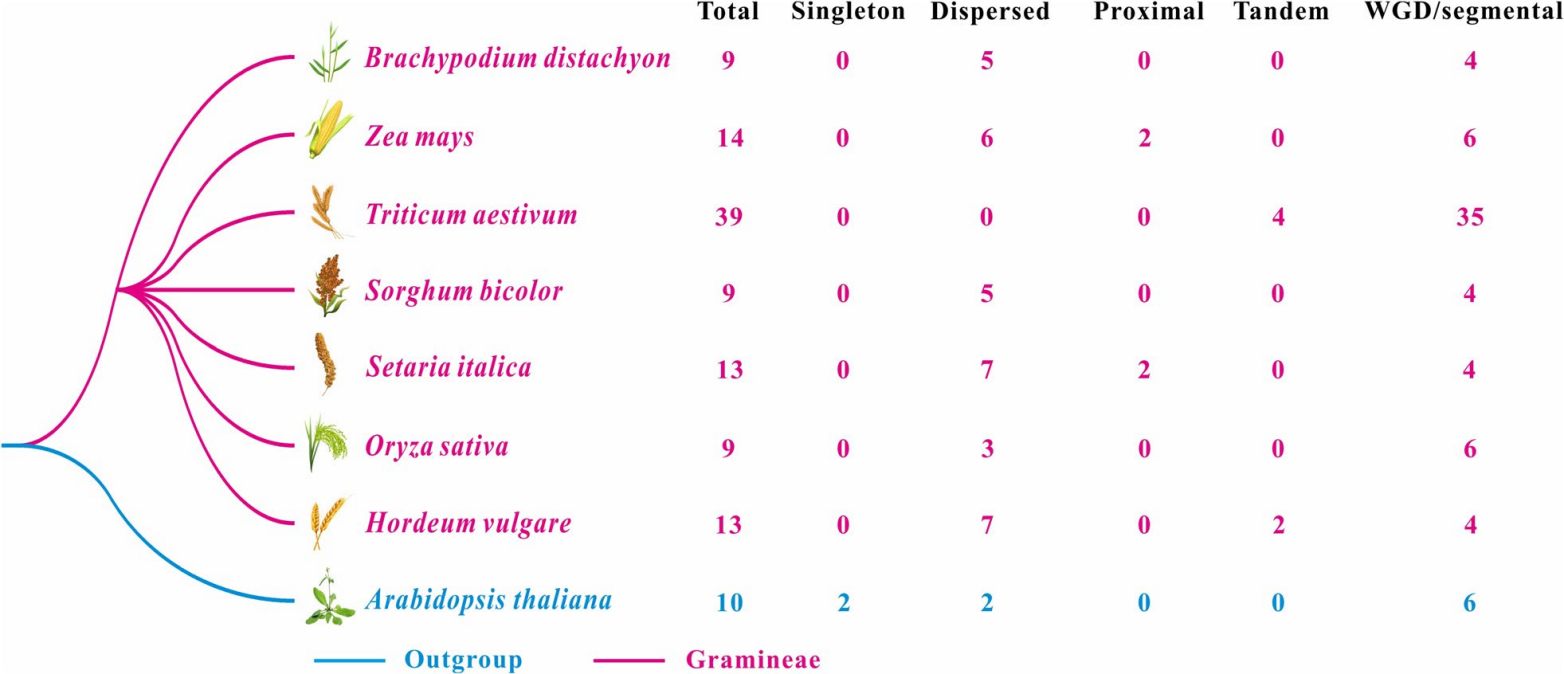
Taxonomic tree of *Z. mays*, *S. bicolor*, *O. sativa*, *B. distachyon*, *S. italica*, *H. vulgare*, *T. aestivum*, and *A. thaliana* as a outgroup species. The total number of *Rboh* genes in five species and *Rboh* genes involved in different duplication-modes were identified. “Total” represents total Rboh protein numbers in each species, while “WGD” represents Whole-genome/Segmental duplication

**Figure. 2:**
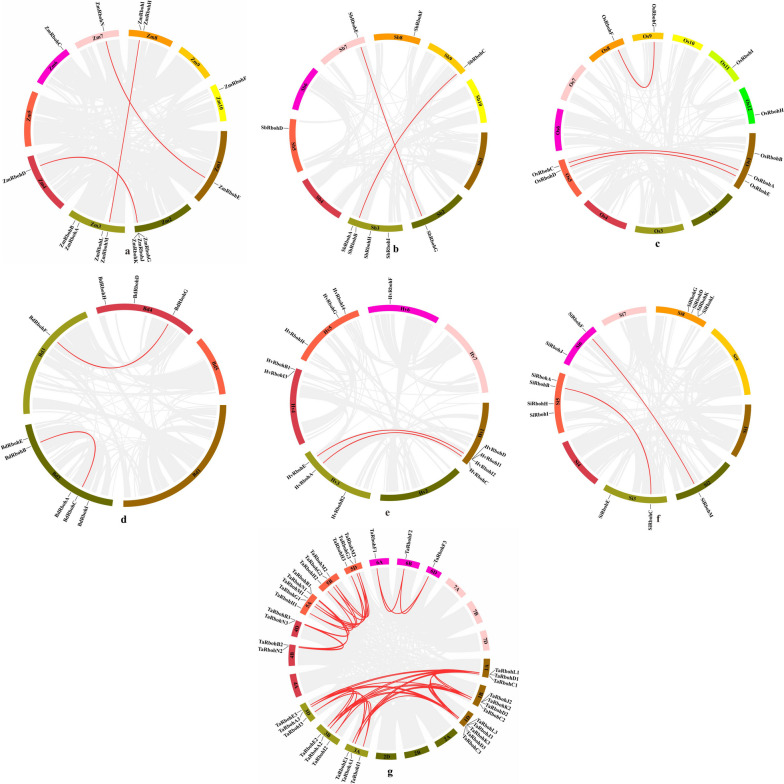
Chromosomal distributions of *Rboh* genes in *Z. mays*, *S. bicolor*, *O. sativa*, *B. distachyon*, *S. italica*, *H. vulgare*, *T. aestivum*. Grey lines represent genome-wide collinear genes in *Z. mays* (**a**), *S. bicolor* (**b**), *O. sativa* (**c**), *B. distachyon* (**d**), *H. vulgare.* (**e**), *S. italica* (**f**) and *T. aestivum* (**g**) while red lines represent collinear *Rboh* genes

A total of 106 *Rboh* genes were identified in the genome of seven gramineous plants, including 14 ZmRboh genes in *Z. mays*, 9 SbRboh genes in *S. bicolor*, 9 BdRboh genes in *B. distachyon*, 9 OsRboh genes in *O. sativa*, 13 SiRboh genes in *S. italica*, 13 HvRboh genes in *H. vulgare*, and 39 TaRboh genes in *T. aestivum* (Fig. 1). The number of *Rboh* family members in the seven grass species ranges from 9 to 14, and *A. thaliana* has similar characteristics, 39 TaRbohs are evenly distributed in the A, B, and D genomes of the *T. aestivum*. These results indicated that the number of *Rboh* family members is not directly proportional to the size of the species genome. The detailed information on *Rboh* genes, including chromosomal distribution, gene length, isoelectric point, and molecular weight, are listed in Table S1. The *Rboh* genes from seven grass plants were distributed unevenly on different chromosomes (Fig. 2). Additionally, the length of all Rboh proteins in seven of the species ranged from 718 aa to 1223 aa, with an average length of 918 aa. The molecular weight of these proteins ranged from 81.76 to 120.57 kDa, with an average of 103.29 kDa. The theoretical pI values of 106 Rboh proteins were larger than 7, indicating that they were alkaline. Finally, all Rboh proteins were predicted to be located on the plasma membrane (PM) (Table S1).

### Collinearity and duplication analysis of *Rboh* genes in *Z. mays* and six other gramineae plants

Multiple duplication modes in angiosperms, including WGD/SD, local duplication (LD) (including tandem and proximal), dispersed duplication, and singleton duplication [30, 31], are the main contributers of gene family expansion. The Rboh family of genes in seven gramineae plants mainly duplicates through WGD/SD and dispersal. However, WGD/SD is the main means of gene family expansion, and the dispersed duplication leads to divergence in gene function. Interestingly, some LD modes were found in *Z. mays, S. italica, H. vulgare*, and *T. aestivum* (Fig. 1 and Table S2).

**Fig. 3:**
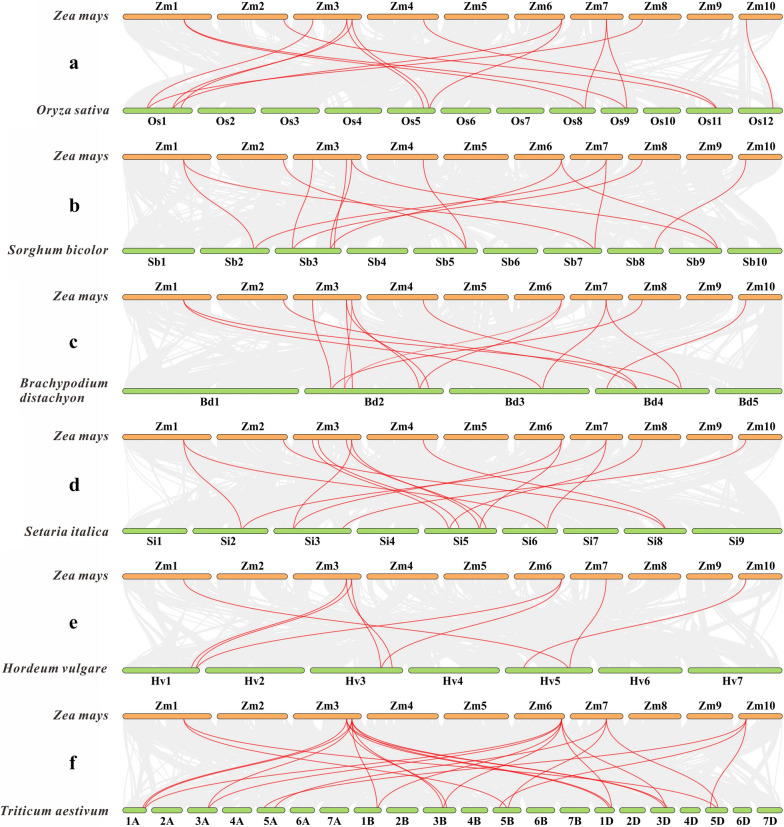
Synteny of *Rboh* genes in the four plants. 10, 10, 12, 5, 9, 7, 21 chromosomes were detected in *Z. mays*, *S. bicolor*, *O. sativa*, *B. distachyon*, *S. italica*, *H. vulgare*, and *T. aestivum*, respectively. Colored boxes indicate chromosome. The inner ring box represents gene density on chromosomes. The syntenic genes were located on the map (represented by colored lines)

All the homologous genes in seven gramineae genomes were identified to analyze the collinearity relationships of the *Rboh* genes (Table S3 and S4). Among seven gramineae plants, we found different putative duplicated gene pairs in each species: 3 gene pairs in *Z. mays* and *O. sativa*; 2 gene pairs in *S. bicolor*, *B. distachyon*, *S. italica*, *and H. vulgare*; 48 gene pairs in *T. aestivum*. All the duplication mods of gene pairs were WGD/SD (Fig. 2 and Table S3). To better understand the evolutionary change of Rboh genes, putative orthologous relationships among all the Rbohs in maize and six other grass plants were established to further elucidate the evolutionary history of the Rboh genes (Fig. 3; Table S4). We identified 11 orthologous gene pairs between maize and other six plants. Among these 11 gene pairs, *ZmRbohB* and *ZmRbohC* were related to *SbRbohB* and *SbRbohC*, *OsRbohA* and *OsRbohC*, *BdRbohB* and *BdRbohC*, *SiRbohB* and *SiRbohC*, *HvRbohA* and *HvRbohC*, and *TaRbohA1/A2/A3* and *TaRbohC1/C2/C3*. On the other hand, *ZmRbohE* and *ZmRbohN* were related to *SbRbohE* and *SbRbohG*, *OsRbohF* and *OsRbohG*, *BdRbohF* and *BdRbohG*, *SiRbohM* and *SiRbohF*, *HvRbohG*, and *TaRbohG1/G2/G3*. These gene pairs were generated using WGD/SD replication, which is perhaps one of the main reasons for the expansion of the *Rboh* gene family. This replication mode may cause functional redundancy among members of the *Rboh* gene family.

**Fig. 4:**
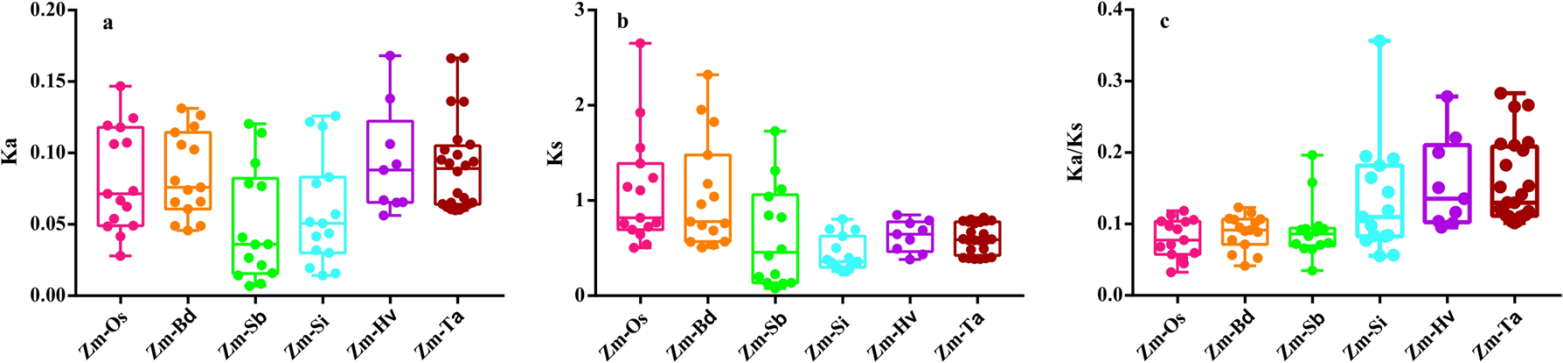
Ka, Ks, and Ka/Ks values of *Rboh* genes in *Z. mays* and six other plants. **a** Ka values of *Rboh* genes pairs between *Z. mays* and *O. sativa*, *Z. mays* and *B. distachyon*, *Z. mays* and *S. bicolor*, *Z. mays* and *S. italica*, *Z. mays* and *H. vulgare*, *Z. mays* and *T. aestivum*, respectively. **b** Ks values of Rboh genes pairs between *Z. mays* and *O. sativa*, *Z. mays* and *B. distachyon*, *Z. mays* and *S. bicolor*, *Z. mays* and *S. italica*, *Z. mays* and *H. vulgare*, *Z. mays* and *T. aestivum*, respectively. **c** Ka/Ks values of Rboh genes pairs between *Z. mays* and *O. sativa*, *Z. mays* and *B. distachyon*, *Z. mays* and *S. bicolor*, *Z. mays* and *S. italica*, *Z. mays* and *H. vulgare*, *Z. mays* and *T. aestivum*, respectively

The evolutionary selection was measured according to the ratio of nonsynonymous-to-synonymous substitutions (Ka/Ks). A pair of sequences with Ka/Ks < 1 implied purifying selection. Ka/Ks = 1 indicated that both genes were drifting neutrally, whereas Ka/Ks > 1 implied a positive or Darwinian selection [32]. According to our data, duplicated pairs among *Z. mays* and six other gramineae plants had different evolutionary rates (Fig. 3 and Table S5). We identified 14 orthologous gene pairs between *Z. mays* and *S. bicolor*, 15 orthologous gene pairs between *Z. mays* and *O. sativa*, *Z. mays* and *B. distachyon*, *Z. mays* and *S. italica*, 9 orthologous gene pairs between *Z. mays* and *H. vulgare*, and 24 orthologous gene pairs between *Z. mays* and *T. aestivum* via MCScanX pipelines. Ka/Ks values between *Z. mays* and six other plants were lower than 1, implying that the gene pairs were mainly under purifying selection. In addition, both Ka and Ks values of Rboh genes in *Z. mays* and *S. bicolor* or *S. italica* were lower than those in *Z. mays* and four other plants, suggesting that the *Rboh* genes evolve more slowly and are conserved in *Z. mays*, *S. bicolor*, and *S. italica* (Fig. 4).

The diversity of the Rboh gene family in maize can be explained using gene and genome duplications. *ZmRbohD* and *ZmRbohK*, *ZmRbohE* and *ZmRbohN*, *ZmRbohI* and *ZmRbohM* in maize genome exhibited WGD/SD duplications, and protein identities were 66.32%, 76.11%, and 92.58%, respectively. The estimation of the divergence time (T) indicated that *ZmRbohD* and *ZmRbohK*, as well as *ZmRbohE* and *ZmRbohN*, were produced from early segmental duplication events ~ 48.50 Mya and 95.48 million years ago Mya (Table S5), respectively. *ZmRbohI* and *ZmRbohM* were produced from recent segmental duplication events 8.59 Mya (Table S5). The maize genome was duplicated 5 to 12 Mya [33], which implied that *ZmRbohI* and *ZmRbohM* were produced during this whole-genome duplication event.

### Phylogenetic and structural analysis of *Rboh* genes in seven surveyed species

**Fig. 5:**
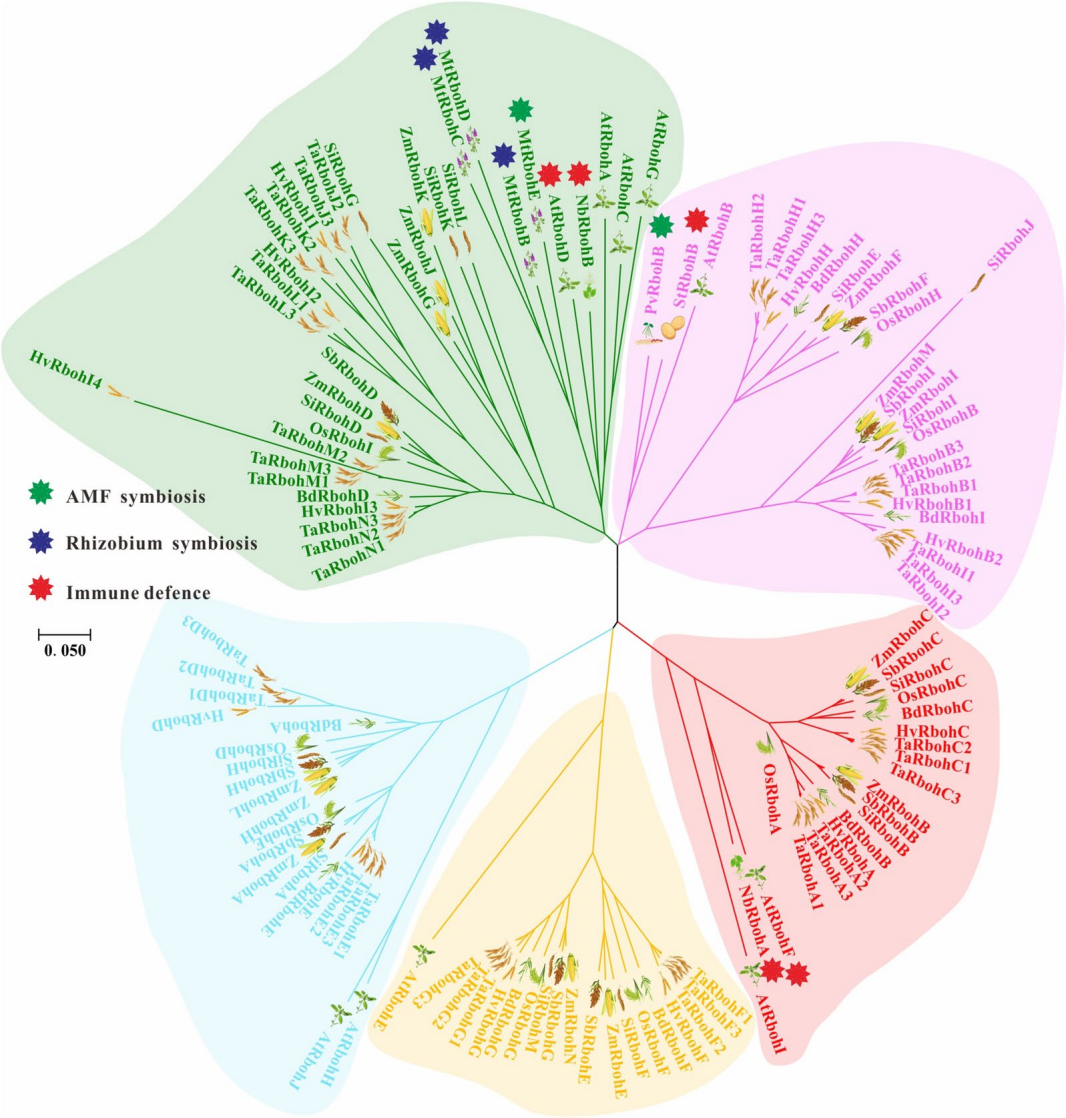
Phylogenetic analysis of *Z. mays*, *S. bicolor*, *O. sativa*, *B. distachyon*, *S. italica*, *H. vulgare*, *T. aestivum* and *A. thaliana*. The tree was constructed via the neighbor-joining method with MEGA 7.0 software. Roman numerals (I-V) indicate the five Rboh subfamilies. Rbohs from *Z. mays* (from ZmRbohA to ZmRbohN), *S. bicolor* (from SbRbohA to SbRbohI), *B. distachyon* (from BdRbohA to BdRbohI), and *O. sativa* (from OsRbohA to OsRbohI), *S. italica* (from SiRbohA to SiRbohM), *H. vulgare* (from HvRbohA to HvRbohI), and *T. aestivum* (from TaRbohA to TaRbohN). The red star indicates that the gene is related to plant immune defense, while the blue and green stars indicate that the gene is related to plant-rhizobia or plant-AM fungal symbiosis

A phylogenetic tree was constructed based on the sequences of Rboh members in *Z. mays*, *O. sativa*, *S. bicolor*, *B. distachyon*, *S. italica*, *H. vulgare*, and *T. aestivum* using MEGA software version 7.0 and the neighbor-joining (NJ) method to elucidate the evolutionary relationships of Rboh genes in seven gramineae plants (Fig. 5). According to the phylogenetic relationship and bootstrap values of branches, five subgroups were identified: subgroup I (AtRbohA, C, D, and G-like), subgroup II (AtRbohB-like), subgroup III (AtRbohE-like), subgroup IV (AtRbohI, F-like), and subgroup V (AtRbohH, J-like). All Rbohs from seven plants were evenly distributed in all five subgroups. However, subgroup I contained the highest numbers of ZmRboh. Generally, *Rboh* genes from *Z. mays* are more closely related to those of *S. bicolor* than other plants, which is consistent the current understanding of the evolutionary history of the plant.

**Fig. 6:**
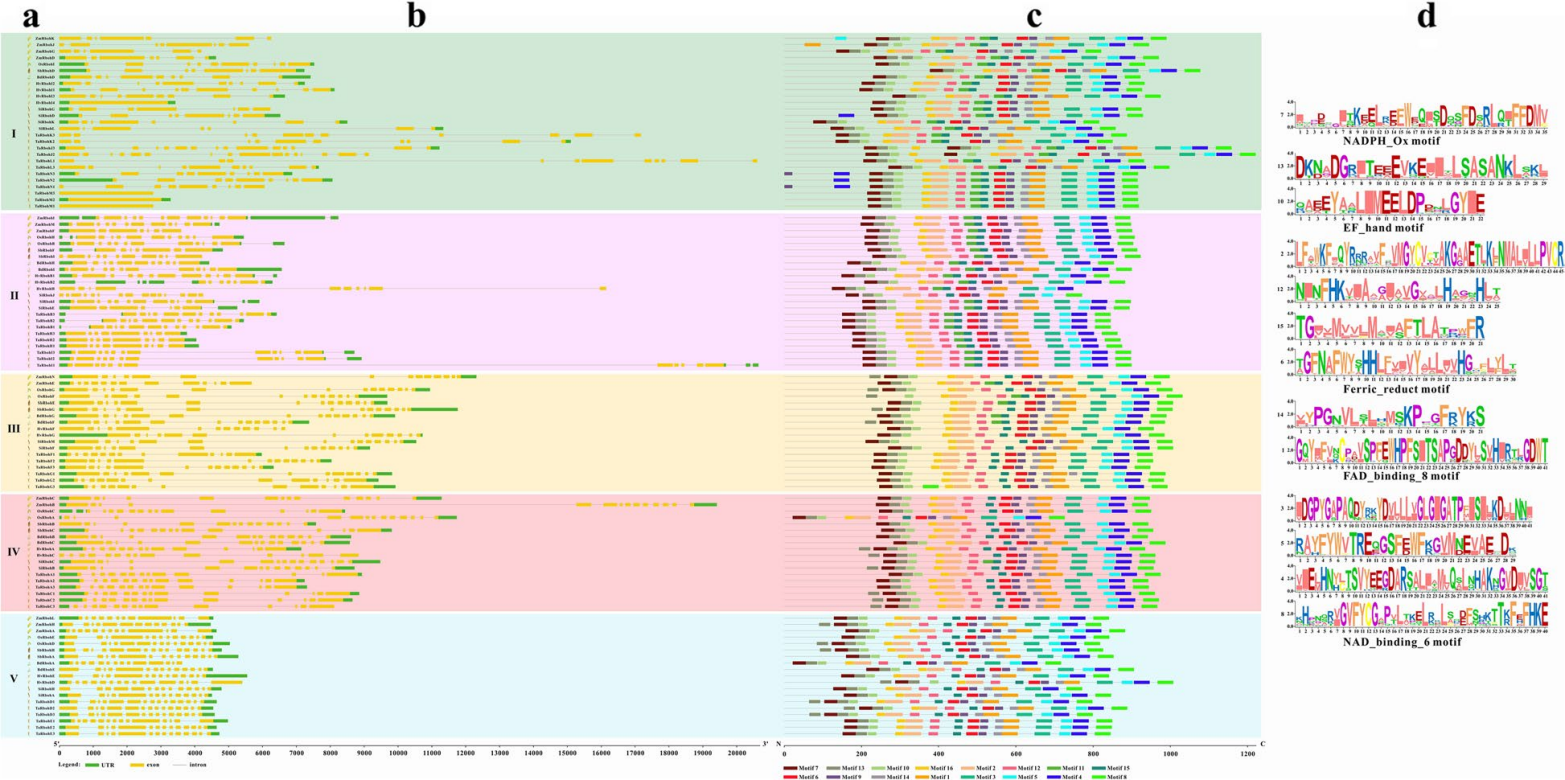
Conserved motif and gene structure of *Rboh* genes. **a** The 106 *Rboh* genes from four plants clustered into five subgroups. **b** Intron/exon structure of *Rboh* genes analyzed by Tbtools. Green and yellow boxes represent UTR and exons, respectively; grey lines represent introns. **c** Sixteen conserved motifs identified via MEME. Different colored boxes with a number represent different motifs. **d** Sequence-logos of conserved motifs. The bit score indicates the content of each position in the amino acid sequence

Subsequently, the exon/intron structure in the coding sequences of *Rboh* genes between and within subgroups was compared to gain further insights into the structural diversity. It was found that most Rbohs in the same subgroups exhibited similar motif features and exon–intron structure (Fig. 6A and B), consistent with their close evolutionary relationship. To further reveal the characteristics of gene structure, 16 putative motifs were predicted in Rbohs using the MEME program (Fig. 6C). Motif 7 was found in the NADPH_Ox domain. Motifs 10 and 13 were found in the EF_hand domain. Ferric_reduct domain was predominant in motifs 2, 6, 12, and 15. The FAD_binding_8 domain contained motifs 1 and 14, and the NAD-binding domain was a combination of motifs 3, 4, 5, and 8. Sequence logos of domain motifs are shown in Fig. 6d. According to the conserved domains, the Rbohs from seven survey species displayed similar motif distribution within each subgroup, indicating a high degree of conservation in each group.

### Tissue specificity expression of Rbohs in seven surveyed species

**Fig. 7:**
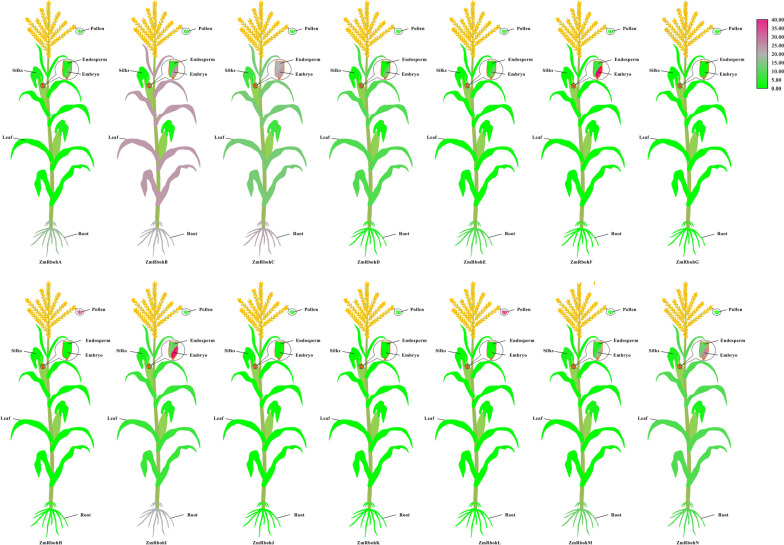
Expression profiles of Rboh genes in *Z. mays*. Color scale at the right represents log2- transformed foldchange values. Purple indicates high expression level; white indicates medium expression level; and green indicates low expression level

Rboh genes were expressed in almost all organs/tissues and different growth stages. We therefore further explored the spatio-temporal expression profiles of Rboh genes in the life cycles of seven gramineae species. As shown in Fig. 7 and Fig. S1, our analyses showed that *Rboh* genes were expressed in specific tissues but in diverse patterns.

In *Z. mays*, *ZmRbohI*, and *ZmRbohF* were mainly expressed in the embryo, whereas *ZmRbohH* and *ZmRbohL* were mainly expressed in mature pollen. *ZmRbohA*, *ZmRbohB*, *ZmRbohC*, and *ZmRbohI* were moderately expressed in the root. None of the Rbohs was expressed in silk, and only *ZmRbohB* was expressed in the leaf. Meanwhile, the expression of *OsRbohB/E/I*, *SbRbohI/C*, *BdRbohI*, *HvRbohI3/B2/C*, and *SiRbohA/B/D/M* was relatively high in the organs/tissues of the tested species. However, a few *Rboh* genes, including *SbRbohH/F*, *BdRbohH/F*, *HvRbohI2/I4*, and *SiRbohG/H*, which were barely expressed in all the survey organs/tissues under normal growth conditions, are perhaps expressed under certain external stimuli, such as biotic and abiotic stresses. Besides, *BdRbohA* was only expressed in the anther of *B. distachyon*. *SbRbohB* was highly expressed in the root of *S. bicolor*, whereas *HvRbohF* was mainly expressed in the internode of *H. vulgare*. Moreover, *OsRbohD* was highly expressed in the anther of *O. sativa*. Generally, these results imply that most members of the Rboh family perform specific functions. In general, the expression of members of the *Rboh* gene family of seven gramineae species differs among tissues/organs and under normal growth conditions, and their expression levels in tissues and organs are relatively low.

### Expression profiles of ZmRboh genes in Responses to maize-AM fungi symbiosis

To explore the potential mechanism by which the expression of *ZmRboh* genes in maize-AM fungi symbiosis is regulated, we identified the cis-elements involved in the activation of symbiosis-related genes in the promoter regions of *ZmRboh*. The Cis-elements involved in AMF induction were detected in 2000 bp upstream regions of *Rboh* genes using the PlantCARE program (Fig. S2). Four fungal induction elements (CTTC, OSEROOTNODULE, NODCON2GM, and W-box) were distributed in the promoter regions of 14 ZmRboh genes. They play an important role in responding to the AM fungus, and their expression could be upregulated by AM fungi. NODCON2GM (CTCTT) element was detected in all 14 *ZmRboh* genes. All four elements existed on the promoter regions of *ZmRbohB*, *ZmRbohI*, and *ZmRbohN*, suggesting that these three *ZmRboh* genes may play a key role in regulating response maize-AM fungi symbiosis. The other *ZmRboh* genes could only detect two or three symbiosis-related elements.

**Fig. 8:**
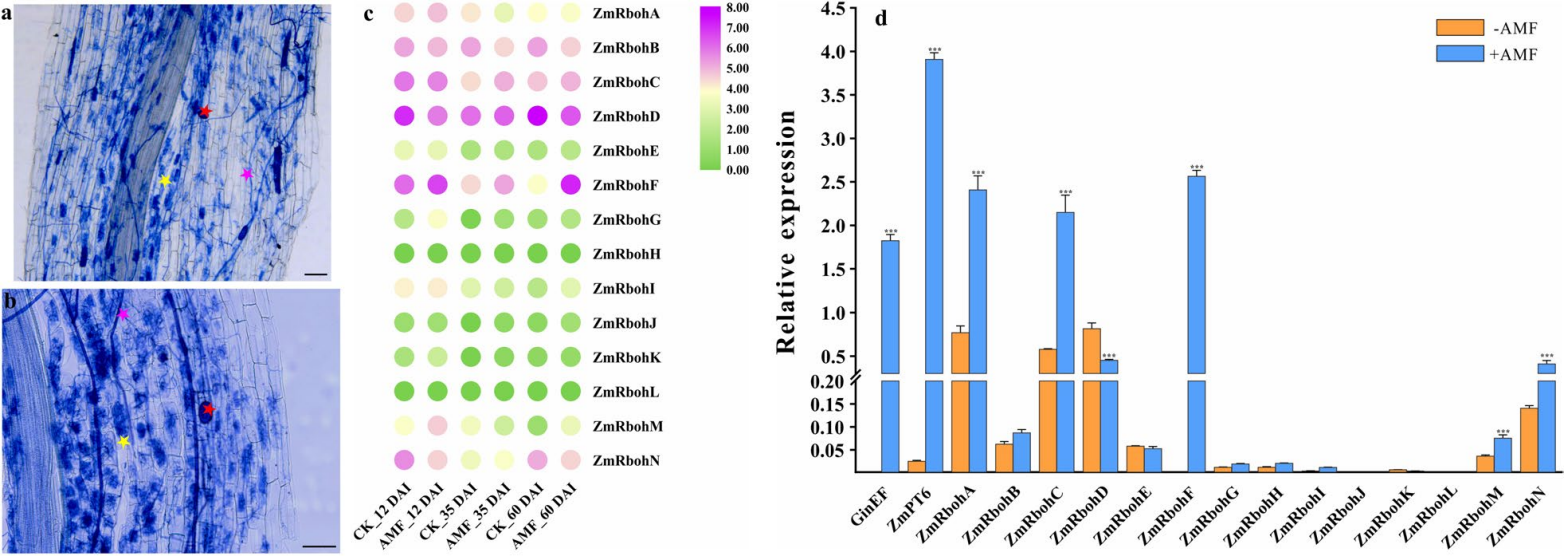
**a** The overall display of the symbiosis relationship between maize roots and AM fungi. **b** Detailed display of symbiosis relationship between maize roots and AM fungi. The purple pentagram represents the hypha, the red pentagram represents vesicle, yellow pentagram shows mature arbuscular, and orange pentagram indicates degraded arbuscular. **c** Expression patterns of ZmRboh genes in root samples in response to arbuscular mycorrhizal fungi. Color scale at the right represents log2-transformed fold-change values. The expression level of each Rboh genes can be estimated based on the scale to the right. Purple indicates high expression level; white indicates medium expression level; and green indicates low expression level. Dpi, day post inoculation. **d** qRT-PCR analysis of ZmRboh genes in root samples in response to Arbuscular Mycorrhizal symbiosis. Maize was grown in nutrient solution containing 50 μM Pi and sampled 60 days after treatment initiation. Data are presented as the means SD (*n* = 3 biological replicates). Asterisks indicate significant differences compared to the control. Student’s *t*-test, *, *P* < 0.05; **, *P* < 0.01; ***, *P* < 0.001. Bars: (a, b) 100 μm

A complete symbiotic system consists of hyphae, vesicles, and arbuscular, these structures recur in the colonized roots and represent a continuous symbiotic process. To better understand symbiotes, we harvested maize inoculated with AM fungi for 60 days and stained their roots (Fig. 8a and b). As expected, we found clear hyphae, vesicles, and arbuscular in maize roots at 60 day post inoculation (dpi). We further investigated how maize Rboh genes change in response to AM fungal inoculation by using transcriptome analysis (Fig. 8c). The expression of Rboh genes showed varies of patterns upon AM fungal inoculation. For instance, *ZmRbohG* and *ZmRbohM* were sensitive to the AM symbiosis and upregulated from 12 dpi, while the expressions of *ZmRbohF* started to increase from 35 dpi and continued to increase at 60 dpi, suggesting these *Rboh* genes may be involved in regulating maize and AM fungi symbiosis. It is notable that *ZmRbohF* may be involved in regulating arbuscular growth and development, given its continuous increased expression at 60 dpi. Consistently, our qRT-PCR analyses confirmed the significant increase *ZmRbohF* in maize roots (Fig. 8d).

### Functional Analysis of *ZmRbohF* in maize-AM fungi symbiosis

**Fig. 9:**
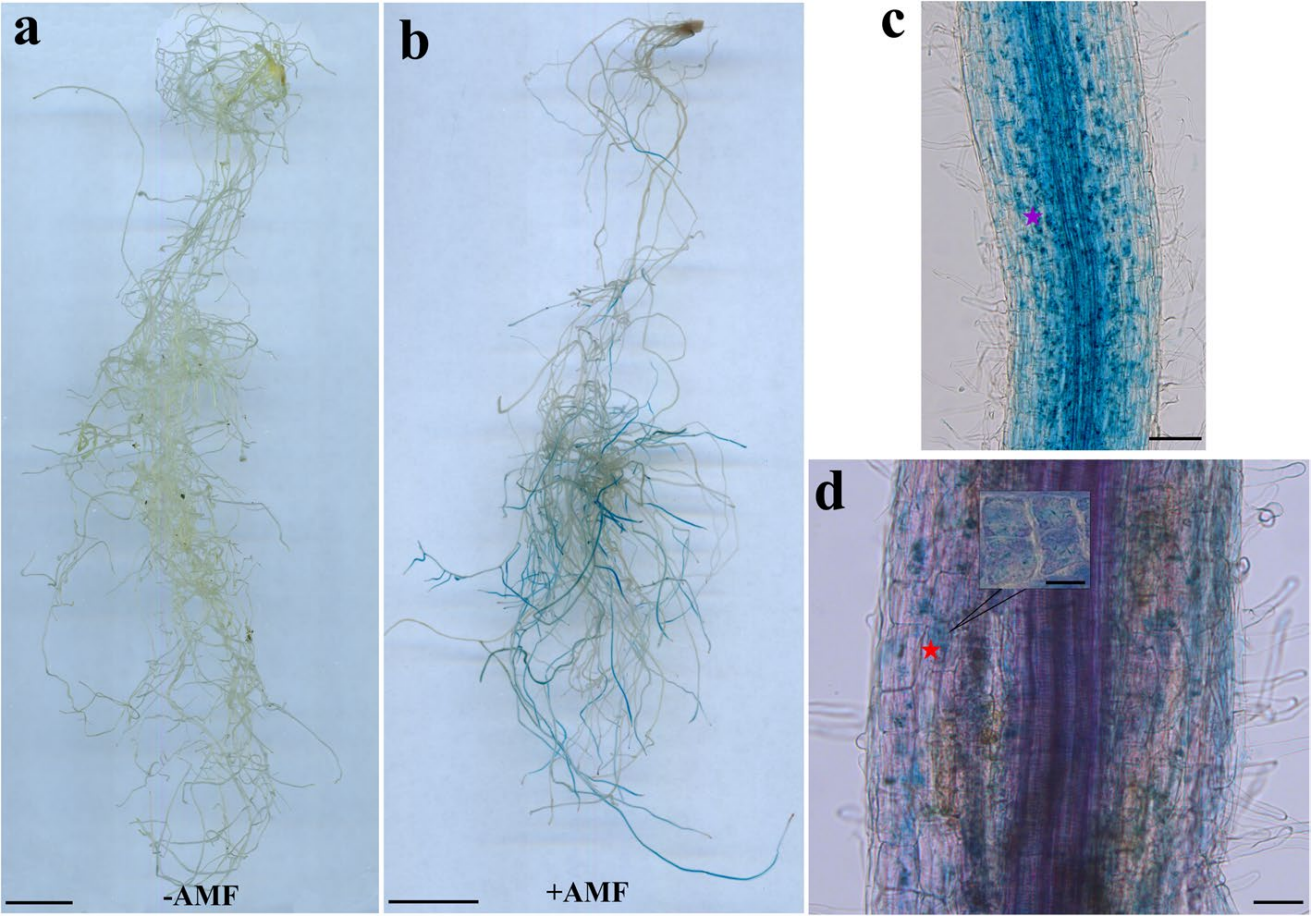
GUS expression induced by AMF in p*ZmRbohF* plants. Star indicate GUS expression positions. **a** Root without inoculated AM fungi stained with X-Gluc dye. **a** Root inoculated AM fungi stained with X-Gluc dye. **c** Microscopy observation of X-Gluc dye stained roots indicating the position of GUS expression, purple stars show the GUS expression regions. **d** Acid fuchsin restaining of the blue roots was observed under microscope, red stars indicate the areas of overlap. Bars: (a, b) 1 cm; (c, d) 100 μm; (d, arbuscular structure) 10 μm

To further examine the potential role of *ZmRbohF* in response to AM fungal inoculation, we firstly assessed the spatial expression pattern of the ZmRbohF promoter during the AM symbiosis in *Lotus japonicus*. A 2000-bp upstream region of the *ZmRbohF* gene was cloned and fused with the chimeric reporter b-glucuronidase (GUS) (p*ZmRbohF*::GUS). Next, the p*ZmRbohF*::GUS reporter construct was transfected into *L. japonicus* using hairy root transformation. The roots of transgenic plants inoculated with AM fungi were stained with X-Gluc dye and Acid Fuchsin to analyze the promoter activity (Fig. 9). The transformed roots inoculated with AM fungi were stained blue using X-Gluc dye to display GUS expression. On the other hand, the roots without AMF were not showed blue after stained (Fig. 9a and b). Microscopy showed that GUS was mainly expressed in the epidermis cells and endothelial cells near the vascular column of symbiotic roots (Fig. 9c). In addition, we stained the roots previously stained blue by X-Gluc dye with acid fuchsin. Microscopic examination revealed that the area of acid fuchsin staining corresponded with the region stained by X-Gluc dye (Fig. 9d). These results indicated that the promoter of *ZmRbohF* can be activated by AM fungi.

**Fig. 10:**
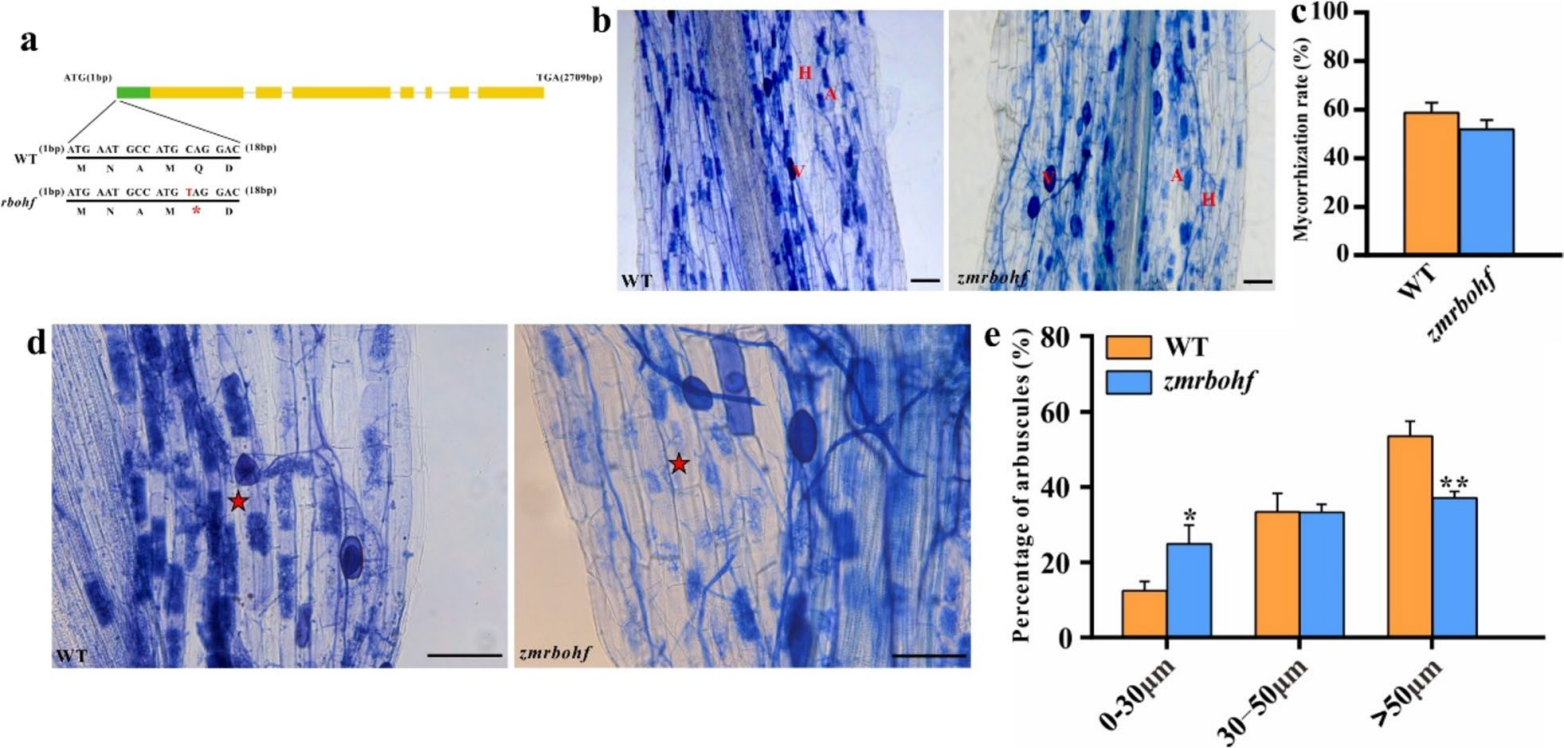
Functional Analysis of ZmRbohF in maize-AM fungi symbiosis. **a** Schematic diagram of z*mrbohf* mutant structure. **b**, **c** Trypan blue staining and colonisation rate statistic in WT and z*mrbohf*. A, arbuscule; V, vesicle; H, hyphae. **d** Details of arbuscule size in WT and mutants. The red star represents the arbuscule. **e** Percentage of arbuscules in WT and z*mrbohf*. Small arbuscule, 0-30μm; middle arbuscule, 30-50μm; mature arbuscule, > 50μm. Data are means ± standard error (SE; n ≥ 3 biological replicates; Student’s *t*-test, *, *p* < 0.05; **, *p* < 0.01). Bars: (b, d) 100 μm

We next sought to test whether *ZmRbohF* is functional relevant to maize-AM fungi symbiosis. To do so, we used a homozygous maize EMS (*zmrbohf*) mutant (Fig. 10a). In brief, the substitution of the 13rd nucleotide (C-T) in the coding region of *ZmRbohF* results in a mutation of glutamine (Q) to a stop codon, leading to premature transcription termination. We inoculated maize roots with AM fungi for 8 weeks, then harvested and stained the roots with Trypan blue. The mycorrhization rates in the roots of WT and *zmrbohf* were similar, suggesting *ZmRbohF* had limited impact on the establishment of maize-AM fungi symbiosis (Fig. 10b and c). Interestingly, we found that the number of mature arbuscules (> 50μm) in the *zmrbohf* were significantly less than that in WT, and *ZmRbohF* loss-of-function caused arbuscules to retain in smaller size (0-30μm) (Fig. 10d and e). Together, these results demonstrated that *ZmRbohF* play critical roles in maize-AM fungi symbiosis through regulating the proper development of arbuscules.

## Discussion

The family of plant *Rboh* genes plays important roles in plant development and stress responses [2, 34]. Studies have shown that the *Rboh* gene family is present in diverse plant species and most members of this family are generally conserved [35–37]. Studies on the *Rboh* gene family have mainly focused on dicot plants, including *A. thaliana* and *M. truncatula* [15, 38]. In this study, 106 Rboh genes were identified from seven species in the gramineae family (Table S1). In many plants analyzed, although the number of Rboh family members varied, the number of genes in the Rboh family was not directly proportional to the genome size [35]. Compared to six other species, 14 members of the Rboh gene family were observed in *Z. mays*. We hypothesize that this may be caused by many chromosome segments repeated in the long evolutionary process of *Z. mays*. A total of 39 Rboh genes were detected in *T. aestivum*. Given that *T. aestivum* is an allohexaploid (genome AABBDD), it is expected that each of the three genomes would contain the same number of genes (homoeologues), the loss and insertion of large segments and the chromosomal rearrangements occur frequently on the chromosomes. These changes might cause gene expression, gene silencing, or loss, and caused the varying levels of Rboh genes in the three subgenomes. All Rboh proteins in the seven species were predicted to be localized in the plasma membrane. This may be attributed to their extremely conservative structure during evolution, which was particularly important for plant environmental adaptation. Consequently, the species always expressed a specific number of proteins.

A gene family generation includes gene duplication resulting from large-scale duplication events (WGD/SD), tandem duplication, and rearrangements at the gene and chromosomal levels [39]. The stress selection analysis revealed that sustained purification selection played a key role in maintaining the number of Rboh genes in maize and six other gramineous plants. Gene duplication and gene family expansion are always followed by functional diversification, and novel genes with diverse functions may play an important role in adapting to new environments [40]. Hence, as shown in Fig. 1, the duplications of all the *Rboh* genes from seven gramineous plants mainly belong to WGD/SD or dispersed group, and this is the main reason for the expansion and functional divergence of the *Rboh* gene family. Surprisingly, some LD modes were found in *Z. mays, S. italica, H. vulgare*, and *T. aestivum*, implying that these genes may have similar functions (Fig. 1). Meanwhile, *ZmRbohD* and *ZmRbohK*, *ZmRbohE* and *ZmRbohN*, and *ZmRbohI* and *ZmRbohM* displayed WGD/SD duplications in the maize genome. Estimation of the divergence time (T) indicated that *ZmRbohI* and *ZmRbohM* were produced from recent segmental duplication events that occurred at approximately 8.59 Mya. The maize genome underwent a duplication event around 5 to 12 Mya [33], implying that *ZmRbohI* and *ZmRbohM* were produced during this whole-genome duplication event (Table S3 and S5). For a better understanding of *Rboh* genes, putative orthologous relationships among all the Rbohs in maize and six other plants were established to further elucidate the evolutionary history of the *Rboh* gene family. We identified 11 orthologous gene pairs between maize and six other plants, and most of these gene pairs are generated using WGD/SD replication, which is perhaps one of the main reasons for the expansion of the Rboh family, and which may cause function redundancy among members of Rboh family (Fig. 3, Table S4, and S5). Ka/Ks values between *Z. mays* and six other plants were less than 1. The selection stress analysis revealed that duplicated gene pairs are mainly under purifying selection. In addition, both Ka and Ks values of Rboh genes were lower in *Z. mays* and *S. bicolor* or *S. italica* than in *Z. mays* and four other plants, implying that the *Rboh* genes evolves more slowly and is conserved in *Z. mays*, *S. bicolor*, and *S. italica* (Fig. 4). The results implied that large-scale duplication contributed significantly to the expansion of *Rboh* genes in maize and the six other plants.

The plant *Rboh* gene catalyzes the production of reactive oxygen species and regulates the symbiosis or immune defense process in the interaction between plants and microorganisms. Phylogenetic analysis was performed to evaluate the evolutionary and functional relationships of *Rboh* genes among seven sampled gramineae plants (Fig. 5). It has been reported that *PvRbohB* and *MtRbohB/C/D/E* participate in regulating plant–microbe symbiosis [4, 15, 26]. *AtRbohD/F*, *NbRbohA/B*, and *StRbohB* mediate plant immune defenses [16, 17], and they belong to subgroups I, II, and IV, respectively. Genes related to symbiosis or immune defense are present in subgroups I and II, the *Rboh* genes from subgroups I and II may have more diverse functions. *Rboh* genes in subgroup IV are more likely to mediate immune defenses. In addition, *ZmRbohD* and *ZmRbohK*, as well as *ZmRbohI* and *ZmRbohM*, generated by WGD/SD may play similar or redundant functions in symbiosis and immune defense. The exon/intron structure revealed that *Rboh* genes in the same subgroups have similar motif features and exon–intron structure, supporting their close evolutionary relationship. Meanwhile, the similarity of all five subgroups ranged from 65.70% to 86.39%, with the high similarity indicating the extremely conservative nature of these genes during evolution. Thus, the high similarity could mean that there may be high functional redundancy among Rboh family members. Thus, further research should be conducted to analyze the evolution and origin of Rboh gene family in gramineous plants.

The current study revealed that the *Rboh* gene participates in the production of reactive oxygen species to regulate the innate immunity of plants during plant-microbial interaction. Thus, the innate immunity role achieves the defense role in the symbiotic relationship. It has been recently reported that *PvRbohB* regulates *Rhizophagus irregularis* colonization in *P. vulgaris* [26, 41]. In *M. truncatula,* another *Rboh* gene, enhances arbuscular mycorrhizal fungal (AMF) colonization [25]. The Rboh-CDPK complex suppresses the innate immunity in *M. truncatula* to enhance symbiotic colonization [15]. In this study, phylogenetic analysis showed that the *Rboh* genes from groups I and II may play important roles in the symbiosis process (Fig. 5). Furthermore, an analysis of cis-acting elements in the promoter region of maize *Rboh* genes was conducted (Fig. S2). The result showed that the promoter region of all the *Rboh* genes contained different numbers of cis-acting elements related to fungal induction. The results of the transcriptome analysis showed that the expression of Rboh genes (*ZmRbohD/G/J/K*) from subgroup I remained unchanged at 12 dpi, 35 dpi, and 60 dpi (Fig. 8c). Moreover, the expression of these genes was relatively low or barely expressed in both roots and leaves (Fig. 7). Since the expression of subgroup I *Rboh* genes remained unchanged after AM fungus inoculation, they are unlikely involved in the symbiosis process. However, they play a role in regulating plant resistance to pathogens. Nonetheless, the expression of *ZmRbohF* from subgroup II was significantly upregulated at 60 dpi (Fig. 8c). This suggests that *ZmRbohF* may regulates symbiotic processes. The result of qRT-PCR showed that the expression of *ZmRbohF* significantly increased at 60 days after AM fungus inoculation (Fig. 8d), suggesting that *ZmRbohF* potentially plays a role in the regulation of the mycorrhizal symbiosis process. After the introduction of p*ZmRbohF*::GUS reporter construct, the hairy roots of *L. japonicus*, after AM fungi inoculation, were stained blue using X-Gluc dye, indicating GUS expression. Microscopy observation of symbiotic roots showed that GUS expression was mainly in the epidermis cell, and endothelial cells near the vascular column. Subsequently, the roots were stained with acid fuchsin following the initial staining with X-Gluc dye, and microscopic observation showed that the acid fuchsin staining region coincided with X-Gluc dye (Fig. 9). These results indicated that the *ZmRbohF* is induced by AM fungi, and it may be involved in the regulation of mazie-AM fungal symbiosis. By employing the *ZmRbohF* mutant, we confirmed that the *ZmRbohF* did not affect the colonization of AM symbiosis. However, *ZmRbohF* loss-of-function led to the significant less number of mature arbuscules (> 50μm) and the more number of small arbuscules (0-30μm). These results indicate that *ZmRbohF* plays an important role in maize-AM fungal symbiosis, mainly by regulating the development of arbuscules.

## Conclusion

In this study, 106 Rboh genes were identified in seven gramineous species, and we performed a comprehensive analysis of the genes, including gene structure, collinearity and duplication, evolutional relationships, promoter sequence, and expression patterns, in the seven species. In addition, the changes in the expression of *ZmRbohA/C/F* in maize inoculated with AM fungi suggested that these genes might be involved in regulating symbiosis processes. Particularly, we selected a candidate gene *ZmRbohF*, the results of RT-qPCR and GUS stained showed *ZmRbohF* was induced by AMF, and *ZmRbohF* loss-of-function affected the ratio of mature arbuscules and small arbuscules, these results showed that *ZmRbohF* was involved in regulating the development of arbuscules in maize. Therefore, this study provides new insight into the evolutionary relationship of Rboh proteins in gramineae plants and the functions they play in the symbiotic relationship between maize and AM fungi.

## Methods

### Identification of *Rboh* genes

Genome and predicted protein sequences of *Z. mays* were obtained from the MaizeGDB database (https://www.maizegdb.org/). *S. bicolor*, *B. distachyon*, and *T. aestivum* were obtained from the gramene database (http://www.gramene.org/). Genome sequences for *O. sativa*, *H. vulgare*, *S. italica*, and *Arabidopsis thaliana* genome sequence was retrieved from Phytozome v12 (https://phytozome.jgi.doe.gov/). To identify Rboh genes in the seven species, reported Rboh protein sequences of Arabidopsis were used to query homologous proteins of *Z. mays*, *S. bicolor*, *O. sativa*, *B. distachyon*, *S. italica*, *H. vulgare*, *T.* *aestivum* using BLASTp with E-values of less than le-5 [42]. These Rboh genes were labeled as ZmRboh, SbRboh, BdRboh, OsRboh, SiRboh, HvRboh, and TaRboh based on previous reports [9, 43, 44]. Hidden Markov Model (HMM) profiles of respiratory burst NADPH oxidase domain (PF08414), Ferric reductase NAD-binding domain (PF08030), FAD-binding domain (PF08022), and Ferric reductase like transmembrane component domain (PF01794) were searched in the Pfam 34.0 database (http://pfam.xfam.org/) and SMART database (http://smart.embl-heidelberg.de/) to select Rboh genes from these seven plants.

### Chromosomal location, phylogenetic analysis, and gene structure

Chromosomal location and gene structure of all Rboh genes were displayed using Tbtools software [45]. The conserved motifs were identified using MEME program Version 5.3.3 (https://meme-suite.org/meme/tools/meme). The analysis of Rboh genes using MEME was performed under specific conditions: (1) allowing any number of repetitions; (2) setting the maximum number of motifs to 16. Further, all motifs detected using MEME were analyzed in Pfam and Smart databases. To investigate the phylogenetic relationship of Rboh proteins among seven gramineous plants, multiple sequence alignments of Rbohs were performed using MEGA 6 [46]. Subsequently, an unrooted phylogenetic tree was constructed using the neighbor-joining (NJ) method. The bootstrap value was 1000.

### Gene duplication and collinearity analysis

MCScanX was used to detect gene duplication events of the Rboh genes [47]. PAL2NAL web server (http://www.bork.embl.de/pal2nal/) was used to calculate nonsynonymous (Ka) and synonymous (Ks) substitution (Ka/Ks) rates [48] using the codeml program of PAML [49]. Moreover, the divergence time of all duplicate pairs was estimated using the following formula: T = Ks / (2 × 9.1 × 10^−9^) × 10^−6^ million years ago (Mya) [50]. The collinearity relationship among the *Rboh* genes of *Z. mays* and six other plants was investigated and displayed using Tbtools software.

### Plant materials and treatments

B73 inbred seeds were obtained from the UniformMu Stock Center (https://www.maizegdb.org/uniformmu). The z*mrbohf* mutant seeds were obtained from MEMD (https://elabcaas.cn/memd/public/index.html#/). The seeds were disinfected with 75% alcohol, then germinated using sterilized germination paper for 4 days. The seedlings were then transferred to AMF spores, vermiculite, perlite, and fine sand pot culture. The seedlings were continuously cultivated with low Pi (50 μM) treatments at the third-leaf stage. The seedlings were grown in a greenhouse at 28 ℃ with a 16 h light/8 h dark photoperiod. The plants were harvested at 60-day post-treatment. The samples were frozen in liquid nitrogen and stored at − 80 °C for subsequent RNA isolation. Meanwhile, the harvested maize roots were fixed with FAA fixing solution overnight. KOH solution (10%) was added to the sample and put the sample on a water bath at 90 °C for 1 h. The roots were treated with a 5% lactic acid solution to make them transparent, then stained with 0.05% trypan blue. Finally, the roots were de-stained using lactic acid-glycerol solution for subsequent microscopic observation, AM colonization quantification was based on[51].

### Mycorrhizal staining

*PZmRbohF*-GUS was transferred into *A. tumefaciens* LBA9402, then grown in YEB solid medium. The verified positive colonies were transferred to YMB solid medium and cultured at 28 °C for two days. The seeds of *Lotus japonicus* were sterilized with 12% NaClO for 10 min, washed thrice with 75% ethanol (1–2 min each wash), followed by washing (3–5 times) with sterile water, (4–5 min each was), then germinated for two days. One hundred pulse roots were then removed from the root tip to the root hair with an aseptic scalpel, and calli were infected with *A. tumefaciens* LBA9402 carrying p*ZmRbohF*-GUS. The infected roots were transferred to B&D medium (Table S7) and cultured in the dark at 28 °C for 24 h, then grown in a light incubator at 23 °C for ~ 3 weeks (8 h photoperiod) before transplanting. The expression of the GUS gene in roots was measured after symbiotic culture with AMF for 6 weeks.

Then harvested the six-weeks-old roots and washed with tap water, the treated roots were put into GUS staining solution (200μL 0.1M K_3_Fe(CN)_6_, 200μL 0.1M K_3_Fe(CN)_6_, 400μL 0.5M PH = 8.0 EDTA Na_2_, 2mL 1M Phosphate Buffer, 200μL 10% TritonX-100, 1mL 20mM X-Gluc, 16mL ddH_2_O), and stained at 37℃ for 24H in a dark environment. Next, the stained roots were further re-dyed with acid fuchsin, a biological dye, which binds to fungal structures. The stained roots were soaked in 10% KOH solution for 30 min, take out the roots from 10% KOH solution and treated with a 5% lactic acid solution for 5 min, subsequently, removed the 5% lactic acid solution and stained with 0.05% acid fuchsin. Finally, the roots were de-stained using lactic acid-glycerolsolution for subsequent microscopic observation.

### Vector construction

The promoter of *ZmRbohF* (p*ZmRbohF*) was used to drive GUS expression of pCAMBIA1301. HindIII and NcoI restriction enzyme sites were incorporated into forward primer 5'-GCAGGCATGCAAGCTTGAGATGAGTGTTTCTGTGCGAG-3' and reverse primer 5'-CTCAGATCTACCATGGTGCGTGAGGCACGCTAGTATGA-3', and the PCR amplification product was ligated to generate the p*ZmRbohF*-GUS plasmid.

### Quantitative Real-Time Polymerase Chain Reaction (qRT-PCR) Analysis

RNA Plus (Takara) was used to extract total RNAs from B73 inoculated with AM fungus for 60 days via the guanidine thiocyanate extraction method. First-strand cDNAs were then synthesized from DNaseI-treated total RNA using reverse transcriptase (Vazyme) and oilgodT primers following the manufacturer’s instructions. RT-PCR was conducted on an Applied Biosystems 7300 using the SYBGREEN kit (Roche) following the manufacturer’s protocol. Relative expression levels were calculated using Actin 1 (J01238) and Alpha-tubulin (X73980) as reference genes, arbuscular mycorrhiza fungal marker gene GinEF [52], maize marker gene ZmPT6 (GRMZM5G881088). qRT-PCR program was conducted as follows: 95 °C for 10 min, followed by 40 cycles at 95 °C for 15 s and 60 °C for 1 min. The qPCR assays had three biological replicates. The primer pairs are listed in Table S6.

### Gene expression profile

The expression data of Rboh genes in tissues of the seven gramineous plants were obtained from the Gramene database (http://www.gramene.org/). Heatmap of the expression was developed using and TBtoolsbased on log_2_-transformed fold-change values.

### Supplementary Information

**Additional file 1:** **Figure S1. **Expression profiles of Rboh genes in* O. sativa* (a), *B. distachyon* (b), *H. vulgare *(c), *S. bicolor* (d) and *S. italica *(e). Color scale at the right represents log2- transformed foldchange values. Purple indicates high expression level; white indicates medium expression level; and green indicates low expression level. DAP Days After Pollination. Color scale at the right represents log2- transformed foldchange values. Purple indicates high expression level; white indicates medium expression level; and green indicates low expression level. DAP Days After Pollination. **Fig. S2. **Regulatory elements on the promoter of ZmRboh genes. Four AM fungal induction-related elements in 2000 bp upstream regions of ZmRboh genes are shown. CTTC (TCTTGTT), OSEROOTNODULE (AAAGAT), NODCON2GM (CTCTT) and W-box (TTGACY).

**Additional file 2:** **Table S1.** Characteristics of Rboh genes in Zea mays, Sorghum bicolor, Brachypodium distachyon, Oryza sativa, Setaria italica, Hordeum vulgare, Triticum aestivum and Arabidopsis thaliana. **Table S2.** Type of Duplicated Rboh genes among seven gramineous plants. **Table S3.** Duplicated Rboh gene pairs Intra-species. **Table S4.** Duplicated Rboh gene pairs among seven gramineous plants. **Table S5.** Estimates of the dates for the large-scale duplication events between Rboh genes in maize and other six plants. **Table S6.** Primers used in this study. **Table S7.** B&D culture medium.

## Data Availability

The sequence information of Rboh family genes in *Z. mays*, *S. bicolor*, *B. distachyon*, *O. sativa*, *S. italica*, *H. vulgare*, *T. aestivum*, and *A. thaliana* was collected from MaizeGDB (https://www.maizegdb.org/), gramene (http://www.gramene.org/), and Phytozome v12 (https://phytozome.jgi.doe.gov/). All other data supporting the results are included within the article and supplementary files.

## Abbreviations

Rboh: Respiratory burst oxidase homolog
AMF: Arbuscular mycorrhizal fungi
ROS: Reactive oxygen species
DAI: Days after inoculation
AA: Amino acid
HMM: Hidden markov model
UTR: Untranslated region
MW: Molecular Weight
pI: Theoretical Isoelectric Point
qRT-PCR: Quantitative real-time polymerase chain reaction
