# Supplementary material for: Systematic analysis of the Rboh gene family in seven gramineous plants and its roles in response to arbuscular mycorrhizal fungi in maize
Source: BMC Plant Biol. 2023 Nov 30;23:603. doi: 10.1186/s12870-023-04571-7 (PMC10688149; doi:10.1186/s12870-023-04571-7)
Supplement: Supplementary file 1 — Additional file 1: Figure S1. Expression profiles of Rboh genes in O. sativa (a), B. distachyon (b), H. vulgare (c), S. bicolor (d) and S. italica (e). Color scale at the right represents log2- transformed foldchange values. Purple indicates high expression level; white indicates medium expression level; and green indicates low expression level. DAP Days After Pollination. Color scale at the right represents log2- transformed foldchange values. Purple indicates high expression level; white indicates medium expression level; and green indicates low expression level. DAP Days After Pollination. Fig. S2. Regulatory elements on the promoter of ZmRboh genes. Four AM fungal induction-related elements in 2000 bp upstream regions of ZmRboh genes are shown. CTTC (TCTTGTT), OSEROOTNODULE (AAAGAT), NODCON2GM (CTCTT) and W-box (TTGACY). [file 12870_2023_4571_MOESM1_ESM.docx]

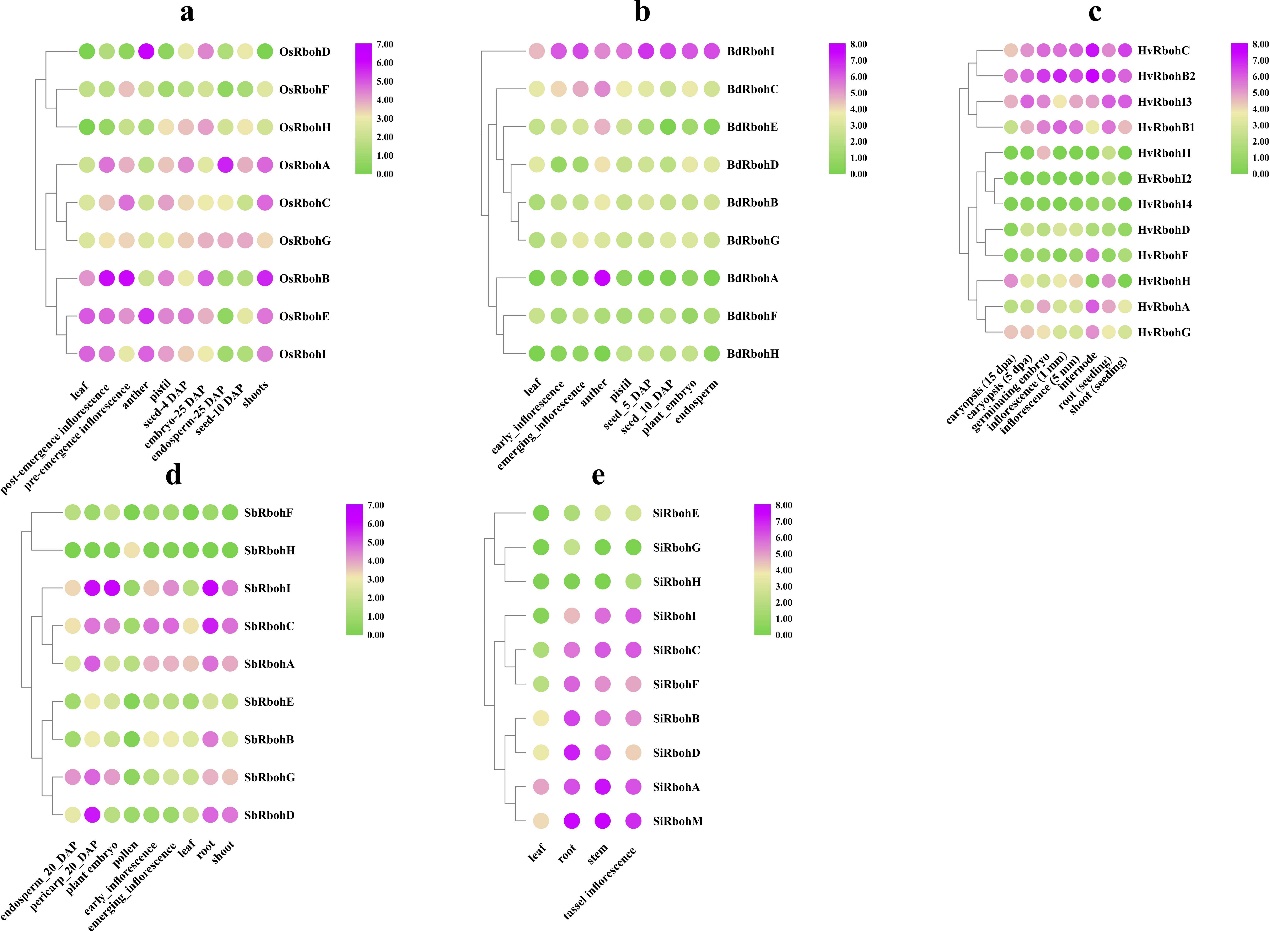


**Figure S1** Expression profiles of Rboh genes in *O. sativa* (a), *B. distachyon* (b), *H. vulgare* (c), *S. bicolor* (d) and *S. italica* (e). Color scale at the right represents log2- transformed foldchange values. Purple indicates high expression level; white indicates medium expression level; and green indicates low expression level. DAP Days After Pollination. Color scale at the right represents log2- transformed foldchange values. Purple indicates high expression level; white indicates medium expression level; and green indicates low expression level. DAP Days After Pollination.

**
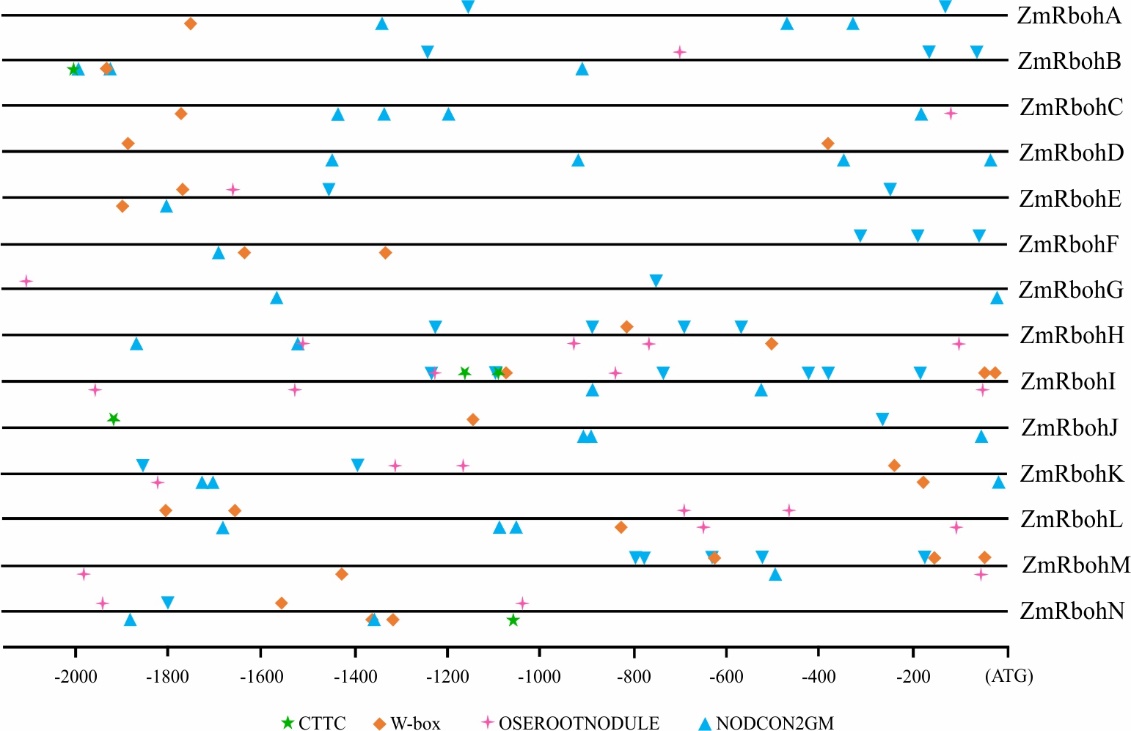
**

**Fig. S2** Regulatory elements on the promoter of ZmRboh genes. Four AM fungal induction-related elements in 2000 bp upstream regions of ZmRboh genes are shown. CTTC (TCTTGTT), OSEROOTNODULE (AAAGAT), NODCON2GM (CTCTT) and W-box (TTGACY).
