# Supplementary material for: Systematic analysis of the Rboh gene family in seven gramineous plants and its roles in response to arbuscular mycorrhizal fungi in maize
Source: BMC Plant Biol. 2023 Nov 30;23:603. doi: 10.1186/s12870-023-04571-7 (PMC10688149; doi:10.1186/s12870-023-04571-7)
Supplement: Supplementary file 2 — Additional file 2: Table S1. Characteristics of Rboh genes in Zea mays, Sorghum bicolor, Brachypodium distachyon, Oryza sativa, Setaria italica, Hordeum vulgare, Triticum aestivum and Arabidopsis thaliana. Table S2. Type of Duplicated Rboh genes among seven gramineous plants. Table S3. Duplicated Rboh gene pairs Intra-species. Table S4. Duplicated Rboh gene pairs among seven gramineous plants. Table S5. Estimates of the dates for the large-scale duplication events between Rboh genes in maize and other six plants. Table S6. Primers used in this study. Table S7. B&D culture medium. [file 12870_2023_4571_MOESM2_ESM.docx]

**Table S1** Characteristics of Rboh genes in Zea mays, Sorghum bicolor, Brachypodium distachyon, Oryza sativa, Setaria italica, Hordeum vulgare, Triticum aestivum and Arabidopsis thaliana

| Species | Gene name | Gene ID | Chromosome location | Gene(bp) | CDS (bp) | Protein size (aa) | Isoelectric Point (pI) | Mw (KDa) | Subcellular localization |
| --- | --- | --- | --- | --- | --- | --- | --- | --- | --- |
| *Zea mays* | ZmRbohA | Zm00001d042961 | 3:184964596-184969233 | 4637 | 2652 | 883 | 9.41 | 99.425 | PM |
|  | ZmRbohB | Zm00001d043543 | 3:203425340-203444747 | 19407 | 2832 | 943 | 9.28 | 106.345 | PM |
|  | ZmRbohC | Zm00001d038762 | 6:164016293-164027578 | 11285 | 2847 | 948 | 9.42 | 106.888 | PM |
|  | ZmRbohD | Zm00001d052653 | 4:196008355-196012956 | 4601 | 2916 | 971 | 9.42 | 108.848 | PM |
|  | ZmRbohE | Zm00001d032079 | 1:211796761-211802432 | 5671 | 2931 | 976 | 9.49 | 108.970 | PM |
|  | ZmRbohF | Zm00001d023859 | 10:25283888-25287483 | 3595 | 2709 | 902 | 9.04 | 100.473 | PM |
|  | ZmRbohG | Zm00001d007430 | 2:231533490-231537684 | 4194 | 2469 | 822 | 9.34 | 92.809 | PM |
|  | ZmRbohH | Zm00001d009349 | 8:57884875-57889340 | 4465 | 2469 | 784 | 9.53 | 88.571 | PM |
|  | ZmRbohI | Zm00001d009248 | 8:47143782-47152014 | 8232 | 2694 | 897 | 9.24 | 101.270 | PM |
|  | ZmRbohJ | Zm00001d007421 | 2:231301639-231307231 | 5592 | 2847 | 948 | 9.26 | 105.990 | PM |
|  | ZmRbohK | Zm00001d007426 | 2:231451417-231457668 | 6251 | 2988 | 995 | 9.21 | 111.958 | PM |
|  | ZmRbohL | Zm00001d040974 | 3:87047940-87052486 | 4546 | 2529 | 842 | 9.33 | 95.015 | PM |
|  | ZmRbohM | Zm00001d040805 | 3:67066176-67071878 | 4722 | 2682 | 893 | 9.40 | 100.774 | PM |
|  | ZmRbohN | Zm00001d020609 | 7:126371894-126384204 | 12310 | 3000 | 999 | 9.24 | 111.316 | PM |
| *Oryza sativa* | OsRbohA | LOC_Os01g53294 | 1:30622671-30634405 | 11735 | 2184 | 727 | 9.05 | 83.411 | PM |
|  | OsRbohB | LOC_Os01g25820 | 1:14621295-14627937 | 6643 | 2718 | 905 | 9.33 | 101.758 | PM |
|  | OsRbohC | LOC_Os05g45210 | 5:26237458-26245892 | 8435 | 2856 | 951 | 9.35 | 107.171 | PM |
|  | OsRbohD | LOC_Os05g38980 | 5:22860152-22865186 | 5035 | 2460 | 819 | 9.18 | 92.350 | PM |
|  | OsRbohE | LOC_Os01g61880 | 1:35813148-35817684 | 4537 | 2532 | 843 | 9.38 | 94.790 | PM |
|  | OsRbohF | LOC_Os08g35210 | 8:22210681-22220352 | 9672 | 3102 | 1033 | 9.84 | 115.015 | PM |
|  | OsRbohG | LOC_Os09g26660 | 9:16186280-16197221 | 10942 | 3024 | 1007 | 9.46 | 112.134 | PM |
|  | OsRbohH | LOC_Os12g35610 | 12:21648123-21653569 | 5447 | 2730 | 909 | 9.20 | 102.122 | PM |
|  | OsRbohI | LOC_Os11g33120 | 11:19588956-19596476 | 7521 | 2811 | 936 | 9.24 | 105.185 | PM |
| *Sorghum bicolor* | SbRbohF | Sobic.008G118700 | 8:53105842-53110669 | 4828 | 2751 | 916 | 8.94 | 101.764 | PM |
|  | SbRbohG | Sobic.002G214200 | 2:60640048-60651809 | 11762 | 3024 | 1007 | 9.11 | 112.077 | PM |
|  | SbRbohC | Sobic.009G206500 | 9:55438002-55447812 | 9811 | 2874 | 957 | 9.43 | 107.461 | PM |
|  | SbRbohB | Sobic.003G287400 | 3:62053854-62061425 | 7572 | 2835 | 944 | 9.29 | 106.568 | PM |
|  | SbRbohA | Sobic.003G347520 | 3:66803346-66808632 | 5287 | 2565 | 854 | 9.42 | 96.547 | PM |
|  | SbRbohH | Sobic.003G175000 | 3:42472858-42477653 | 4796 | 2484 | 827 | 9.38 | 93.653 | PM |
|  | SbRbohI | Sobic.003G161500 | 3:19272846-19277057 | 4212 | 2775 | 924 | 9.26 | 103.925 | PM |
|  | SbRbohD | Sobic.005G139700 | 5:59440994-59448226 | 7233 | 3240 | 1079 | 9.57 | 120.570 | PM |
|  | SbRbohE | Sobic.007G148300 | 7:57917055-57926738 | 9684 | 3027 | 1008 | 9.60 | 112.949 | PM |
| *Brachypodium distachyon* | BdRbohI | BRADI_2g12790v3 | 2:11162986-11169546 | 6561 | 2706 | 901 | 9.24 | 101.582 | PM |
|  | BdRbohE | BRADI_2g54240v3 | 2:52983795-52988328 | 4534 | 2724 | 907 | 9.51 | 101.554 | PM |
|  | BdRbohA | BRADI_2g22820v3 | 2:20315922-20319541 | 3620 | 2157 | 718 | 8.61 | 81.755 | PM |
|  | BdRbohC | BRADI_2g19090v3 | 2:16790284-16798858 | 8575 | 2970 | 989 | 9.28 | 110.417 | PM |
|  | BdRbohB | BRADI_2g49040v3 | 2:48973670-48982283 | 8614 | 2832 | 943 | 9.38 | 106.370 | PM |
|  | BdRbohF | BRADI_3g37530v3 | 3:39777667-39785036 | 7370 | 2964 | 987 | 9.43 | 109.808 | PM |
|  | BdRbohD | BRADI_4g17020v3 | 4:17969038-17976448 | 7411 | 2775 | 924 | 9.35 | 104.290 | PM |
|  | BdRbohG | BRADI_4g31130v3 | 4:36803159-36813070 | 9912 | 2976 | 991 | 9.42 | 111.113 | PM |
|  | BdRbohH | BRADI_4g05540v3 | 4:4661124-4665542 | 4419 | 2568 | 855 | 9.05 | 97.129 | PM |
| *Hordeum vulgare* | HvRbohD | HORVU1Hr1G071340 | 1:492785654-492791048 | 5394 | 3036 | 1011 | 9.62 | 113.611 | PM |
|  | HvRbohI1 | HORVU1Hr1G072140 | 1:496197193-496205312 | 8119 | 2790 | 929 | 9.22 | 105.726 | PM |
|  | HvRbohI2 | HORVU1Hr1G072160 | 1:496271618-496278858 | 7240 | 2808 | 935 | 9.39 | 104.477 | PM |
|  | HvRbohC | HORVU1Hr1G081950 | 1:528355863-528364696 | 8833 | 2907 | 968 | 9.27 | 108.232 | PM |
|  | HvRbohB2 | HORVU3Hr1G037600 | 3:213937393-213943684 | 6291 | 2658 | 885 | 9.19 | 99.553 | PM |
|  | HvRbohA | HORVU3Hr1G069780 | 3:528606381-528613529 | 7148 | 2814 | 937 | 9.24 | 105.837 | PM |
|  | HvRbohE | HORVU3Hr1G087210 | 3:620566836-620572379 | 5543 | 2601 | 866 | 9.45 | 95.014 | PM |
|  | HvRbohI3 | HORVU4Hr1G081670 | 4:620019028-620025685 | 6657 | 2934 | 977 | 9.39 | 109.763 | PM |
|  | HvRbohB1 | HORVU4Hr1G086500 | 4:634474440-634480861 | 6421 | 2538 | 845 | 9.10 | 94.963 | PM |
|  | HvRbohG | HORVU5Hr1G062490 | 5:486161210-486171931 | 10721 | 2964 | 987 | 9.25 | 110.498 | PM |
|  | HvRbohH | HORVU5Hr1G024550 | 5:132503773-132519904 | 16131 | 2322 | 773 | 8.92 | 87.634 | PM |
|  | HvRbohI4 | HORVU5Hr1G078630 | 5:555446291-555449711 | 3420 | 2889 | 962 | 10.30 | 108.042 | PM |
|  | HvRbohF | HORVU6Hr1G035970 | 6:175818335-175824999 | 6664 | 2880 | 959 | 9.24 | 106.519 | PM |
| *Setaria italica* | SiRbohA | Seita.5G372100 | 5:40837073-40842276 | 4507 | 2547 | 848 | 9.49 | 95.934 | PM |
|  | SiRbohB | Seita.5G307900 | 5:36098874-36107412 | 8716 | 2877 | 958 | 9.27 | 107.745 | PM |
|  | SiRbohC | Seita.3G170600 | 3:12600525-12609637 | 9473 | 2892 | 963 | 9.34 | 108.248 | PM |
|  | SiRbohD | Seita.8G141900 | 8:27579917-27585588 | 6521 | 2796 | 931 | 9.36 | 104.858 | PM |
|  | SiRbohE | Seita.3G343100 | 3:44180458-44185472 | 5252 | 2691 | 896 | 9.37 | 99.770 | PM |
|  | SiRbohF | Seita.6G170500 | 6:29425772-29434807 | 9178 | 3030 | 1009 | 9.59 | 111.837 | PM |
|  | SiRbohG | Seita.8G129100 | 8:24779720-24785677 | 6235 | 2784 | 927 | 9.16 | 104.130 | PM |
|  | SiRbohH | Seita.5G182900 | 5:23060050-23064529 | 4787 | 2322 | 773 | 9.44 | 87.577 | PM |
|  | SiRbohI | Seita.5G164400 | 5:16065359-16071023 | 5902 | 2700 | 899 | 9.21 | 101.565 | PM |
|  | SiRbohJ | Seita.6G075000 | 6:6602195-6606440 | 4246 | 2319 | 772 | 8.95 | 87.723 | PM |
|  | SiRbohK | Seita.8G184400 | 8:32972926-32981394 | 8468 | 2460 | 819 | 8.46 | 93.918 | PM |
|  | SiRbohL | Seita.8G185000 | 8:33066134-33077469 | 11335 | 2577 | 858 | 8.79 | 97.400 | PM |
|  | SiRbohM | Seita.2G216800 | 2:31860022-31870554 | 10532 | 3033 | 1010 | 9.21 | 111.943 | PM |
| *Triticum  aestivum* | TaRbohA1 | TraesCS3A02G280200 | 3A:509419282-509428289 | 8938 | 2931 | 976 | 9.42 | 109.814 | PM |
|  | TaRbohA2 | TraesCS3B02G314000 | 3B:504979749-504986950 | 7242 | 2835 | 944 | 9.26 | 106.663 | PM |
|  | TaRbohA3 | TraesCS3D02G279900 | 3D:388330898-388338213 | 7316 | 2841 | 946 | 9.28 | 106.852 | PM |
|  | TaRbohB1 | TraesCS5A02G527600 | 5A:688137629-688142671 | 5083 | 2544 | 847 | 9.18 | 95.219 | PM |
|  | TaRbohB2 | TraesCS4B02G358800 | 4B:649469807-649475248 | 5442 | 2544 | 847 | 9.19 | 95.330 | PM |
|  | TaRbohB3 | TraesCS4D02G352200 | 4D:503615478-503621888 | 6411 | 2544 | 847 | 9.22 | 95.416 | PM |
|  | TaRbohC1 | TraesCS1A02G347700 | 1A:533447254-533456110 | 8857 | 2886 | 961 | 9.23 | 107.920 | PM |
|  | TaRbohC2 | TraesCS1B02G362200 | 1B:591109334-591117986 | 8653 | 2919 | 972 | 9.23 | 108.913 | PM |
|  | TaRbohC3 | TraesCS1D02G350600 | 1D:436189306-436197416 | 8111 | 2907 | 968 | 9.23 | 108.569 | PM |
|  | TaRbohD1 | TraesCS1A02G290700 | 1A:487088736-487093379 | 4644 | 2403 | 800 | 9.06 | 90.833 | PM |
|  | TaRbohD2 | TraesCS1B02G300000 | 1B:520781252-520785799 | 4548 | 2673 | 890 | 9.16 | 100.557 | PM |
|  | TaRbohD3 | TraesCS1D02G289300 | 1D:387423339-387427925 | 4587 | 2403 | 800 | 9.06 | 91.043 | PM |
|  | TaRbohE1 | TraesCS3A02G354200 | 3A:601556249-601561226 | 4978 | 2556 | 851 | 9.44 | 95.748 | PM |
|  | TaRbohE2 | TraesCS3B02G386600 | 3B:607379942-607384583 | 4642 | 2553 | 850 | 9.47 | 95.660 | PM |
|  | TaRbohE3 | TraesCS3D02G347900 | 3D:459463128-459467853 | 4726 | 2544 | 847 | 9.45 | 95.315 | PM |
|  | TaRbohF1 | TraesCS6A02G180600 | 6A:204738949-204744921 | 5973 | 2871 | 956 | 9.26 | 106.597 | PM |
|  | TaRbohF2 | TraesCS6B02G205000 | 6B:255665332-255673360 | 8029 | 2874 | 957 | 9.04 | 106.848 | PM |
|  | TaRbohF3 | TraesCS6D02G164500 | 6D:143641434-143647754 | 6321 | 2853 | 950 | 9.36 | 106.360 | PM |
|  | TaRbohG1 | TraesCS5A02G211800 | 5A:427065400-427075221 | 9822 | 2970 | 989 | 9.23 | 110.951 | PM |
|  | TaRbohG2 | TraesCS5B02G212100 | 5B:382769907-382779292 | 9426 | 2958 | 985 | 9.19 | 110.737 | PM |
|  | TaRbohG3 | TraesCS5D02G222100 | 5D:329610128-329620046 | 9919 | 2985 | 994 | 9.19 | 111.265 | PM |
|  | TaRbohH1 | TraesCS5A02G093600 | 5A:130083471-130087583 | 4113 | 2634 | 877 | 9.10 | 98.755 | PM |
|  | TaRbohH2 | TraesCS5B02G099700 | 5B:132600898-132604936 | 4039 | 2622 | 873 | 9.16 | 98.179 | PM |
|  | TaRbohH3 | TraesCS5D02G105900 | 5D:119706812-119710572 | 3761 | 2598 | 865 | 9.11 | 97.360 | PM |
|  | TaRbohI1 | TraesCS3A02G182900 | 3A:212112431-212133056 | 20626 | 2706 | 901 | 9.35 | 101.630 | PM |
|  | TaRbohI2 | TraesCS3B02G212900 | 3B:252588322-252597242 | 8921 | 2703 | 900 | 9.34 | 101.457 | PM |
|  | TaRbohI3 | TraesCS3D02G187300 | 3D:173707875-173716585 | 8711 | 2703 | 900 | 9.34 | 101.437 | PM |
|  | TaRbohJ2 | TraesCS1B02G295200 | 1B:513155891-513165024 | 9134 | 3672 | 1223 | 9.00 | 139.421 | PM |
|  | TaRbohJ3 | TraesCS1D02G284900 | 1D:382916208-382927430 | 11223 | 3483 | 1160 | 8.88 | 131.928 | PM |
|  | TaRbohK2 | TraesCS1B02G295300 | 1B:513464187-513479278 | 15092 | 2559 | 852 | 9.30 | 96.516 | PM |
|  | TaRbohK3 | TraesCS1D02G285000 | 1D:383156727-383173887 | 17161 | 2727 | 908 | 8.91 | 102.206 | PM |
|  | TaRbohL1 | TraesCS1A02G285700 | 1A:482794568-482815155 | 20588 | 2805 | 934 | / | / | PM |
|  | TaRbohL3 | TraesCS1D02G284800 | 1D:382892913-382900563 | 11979 | 3000 | 999 | 9.18 | 110.812 | PM |
|  | TaRbohM1 | TraesCS5A02G301700 | 5A:510541847-510544609 | 2763 | 2763 | 920 | 9.33 | 102.801 | PM |
|  | TaRbohM2 | TraesCS5B02G299000 | 5B:482288012-482291300 | 3289 | 2757 | 918 | 9.24 | 102.744 | PM |
|  | TaRbohM3 | TraesCS5D02G306400 | 5D:402950923-402953676 | 2754 | 2754 | 917 | 9.28 | 102.638 | PM |
|  | TaRbohN1 | TraesCS5A02G499900 | 5A:666228791-666234841 | 6051 | 2760 | 919 | 9.28 | 103.462 | PM |
|  | TaRbohN2 | TraesCS4B02G327800 | 4B:618587679-618595742 | 8064 | 2754 | 917 | 9.28 | 103.529 | PM |
|  | TaRbohN3 | TraesCS4D02G324800 | 4D:485382384-485389255 | 6872 | 2757 | 918 | 9.28 | 103.538 | PM |
| *Arabidopsis  thaliana* | AtRbohA | At5g07390.1 | 5:2335895-2339913 | 4019 | 2709 | 902 | 9.26 | 102.935 | PM |
|  | AtRbohB | At1g09090.2 | 1:2932739-2936586 | 3848 | 2532 | 843 | 9.26 | 96.390 | PM |
|  | AtRbohC | At5g51060.1 | 5:20757199-20762461 | 5148 | 2718 | 905 | 9.50 | 102.518 | PM |
|  | AtRbohD | At5g47910.1 | 5:19397041-19402061 | 4619 | 2766 | 921 | 9.27 | 103.908 | PM |
|  | AtRbohE | At1g19230.2 | 1:6643942-6649375 | 5034 | 2805 | 934 | 8.95 | 107.702 | PM |
|  | AtRbohF | At1g64060.1 | 1:23769774-23776984 | 7158 | 2835 | 944 | 9.23 | 108.418 | PM |
|  | AtRbohG | At4g25090.1 | 4:12878667-12883805 | 4825 | 2550 | 849 | 9.10 | 96.862 | PM |
|  | AtRbohH | At5g60010.1 | 5:24160270-24165052 | 4300 | 2661 | 886 | 9.25 | 100.627 | PM |
|  | AtRbohI | At4g11230.1 | 4:6840473-6845627 | 5101 | 2826 | 941 | 8.71 | 106.952 | PM |
|  | AtRbohJ | At3g45810.1 | 3:16832726-16837792 | 4687 | 2739 | 912 | 9.48 | 102.936 | PM |

**Table S2** Type of Duplicated Rboh genes among seven gramineous plants.

| Species | Gene name | Gene ID | Duplicate type |
| --- | --- | --- | --- |
| *Zea mays* | ZmRbohA | Zm00001d042961 | 1 |
|  | ZmRbohB | Zm00001d043543 | 1 |
|  | ZmRbohC | Zm00001d038762 | 1 |
|  | ZmRbohD | Zm00001d052653 | 4 |
|  | ZmRbohE | Zm00001d032079 | 4 |
|  | ZmRbohF | Zm00001d023859 | 1 |
|  | ZmRbohG | Zm00001d007430 | 2 |
|  | ZmRbohH | Zm00001d009349 | 1 |
|  | ZmRbohI | Zm00001d009248 | 4 |
|  | ZmRbohJ | Zm00001d007421 | 2 |
|  | ZmRbohK | Zm00001d007426 | 4 |
|  | ZmRbohL | Zm00001d040974 | 1 |
|  | ZmRbohM | Zm00001d040805 | 4 |
|  | ZmRbohN | Zm00001d020609 | 4 |
| *Oryza sativa* | OsRbohA | LOC_Os01g53294 | 4 |
|  | OsRbohB | LOC_Os01g25820 | 1 |
|  | OsRbohC | LOC_Os05g45210 | 4 |
|  | OsRbohD | LOC_Os05g38980 | 4 |
|  | OsRbohE | LOC_Os01g61880 | 4 |
|  | OsRbohF | LOC_Os08g35210 | 4 |
|  | OsRbohG | LOC_Os09g26660 | 4 |
|  | OsRbohH | LOC_Os12g35610 | 1 |
|  | OsRbohI | LOC_Os11g33120 | 1 |
| *Sorghum bicolor* | SbRbohF | Sobic.008G118700 | 1 |
|  | SbRbohG | Sobic.002G214200 | 4 |
|  | SbRbohC | Sobic.009G206500 | 4 |
|  | SbRbohB | Sobic.003G287400 | 4 |
|  | SbRbohA | Sobic.003G347520 | 1 |
|  | SbRbohH | Sobic.003G175000 | 1 |
|  | SbRbohI | Sobic.003G161500 | 1 |
|  | SbRbohD | Sobic.005G139700 | 1 |
|  | SbRbohE | Sobic.007G148300 | 4 |
| *Brachypodium distachyon* | BdRbohI | BRADI_2g12790v3 | 1 |
|  | BdRbohE | BRADI_2g54240v3 | 1 |
|  | BdRbohA | BRADI_2g22820v3 | 1 |
|  | BdRbohC | BRADI_2g19090v3 | 4 |
|  | BdRbohB | BRADI_2g49040v3 | 4 |
|  | BdRbohF | BRADI_3g37530v3 | 4 |
|  | BdRbohD | BRADI_4g17020v3 | 1 |
|  | BdRbohG | BRADI_4g31130v3 | 4 |
|  | BdRbohH | BRADI_4g05540v3 | 1 |
| *Arabidopsis thaliana* | AtRbohA | At5g07390.1 | 1 |
|  | AtRbohB | At1g09090.2 | 0 |
|  | AtRbohC | At5g51060.1 | 4 |
|  | AtRbohD | At5g47910.1 | 1 |
|  | AtRbohE | At1g19230.2 | 0 |
|  | AtRbohF | At1g64060.1 | 4 |
|  | AtRbohG | At4g25090.1 | 4 |
|  | AtRbohH | At5g60010.1 | 4 |
|  | AtRbohI | At4g11230.1 | 4 |
|  | AtRbohJ | At3g45810.1 | 4 |
| *Hordeum vulgare* | HvRbohD | HORVU1Hr1G071340 | 4 |
|  | HvRbohI1 | HORVU1Hr1G072140 | 3 |
|  | HvRbohI2 | HORVU1Hr1G072160 | 3 |
|  | HvRbohC | HORVU1Hr1G081950 | 4 |
|  | HvRbohB2 | HORVU3Hr1G037600 | 1 |
|  | HvRbohA | HORVU3Hr1G069780 | 4 |
|  | HvRbohE | HORVU3Hr1G087210 | 4 |
|  | HvRbohI3 | HORVU4Hr1G081670 | 1 |
|  | HvRbohB1 | HORVU4Hr1G086500 | 1 |
|  | HvRbohG | HORVU5Hr1G062490 | 1 |
|  | HvRbohH | HORVU5Hr1G024550 | 1 |
|  | HvRbohI4 | HORVU5Hr1G078630 | 1 |
|  | HvRbohF | HORVU6Hr1G035970 | 1 |
| *Setaria italica* | SiRbohA | Seita.5G372100 | 1 |
|  | SiRbohB | Seita.5G307900 | 4 |
|  | SiRbohC | Seita.3G170600 | 4 |
|  | SiRbohD | Seita.8G141900 | 1 |
|  | SiRbohE | Seita.3G343100 | 1 |
|  | SiRbohF | Seita.6G170500 | 4 |
|  | SiRbohG | Seita.8G129100 | 1 |
|  | SiRbohH | Seita.5G182900 | 1 |
|  | SiRbohI | Seita.5G164400 | 1 |
|  | SiRbohJ | Seita.6G075000 | 1 |
|  | SiRbohK | Seita.8G184400 | 2 |
|  | SiRbohL | Seita.8G185000 | 2 |
|  | SiRbohM | Seita.2G216800 | 4 |
| *Triticum aestivum* | TaRbohA1 | TraesCS3A02G280200 | 4 |
|  | TaRbohA2 | TraesCS3B02G314000 | 4 |
|  | TaRbohA3 | TraesCS3D02G279900 | 4 |
|  | TaRbohB1 | TraesCS5A02G527600 | 4 |
|  | TaRbohB2 | TraesCS4B02G358800 | 4 |
|  | TaRbohB3 | TraesCS4D02G352200 | 4 |
|  | TaRbohC1 | TraesCS1A02G347700 | 4 |
|  | TaRbohC2 | TraesCS1B02G362200 | 4 |
|  | TaRbohC3 | TraesCS1D02G350600 | 4 |
|  | TaRbohD1 | TraesCS1A02G290700 | 4 |
|  | TaRbohD2 | TraesCS1B02G300000 | 4 |
|  | TaRbohD3 | TraesCS1D02G289300 | 4 |
|  | TaRbohE1 | TraesCS3A02G354200 | 4 |
|  | TaRbohE2 | TraesCS3B02G386600 | 4 |
|  | TaRbohE3 | TraesCS3D02G347900 | 4 |
|  | TaRbohF1 | TraesCS6A02G180600 | 4 |
|  | TaRbohF2 | TraesCS6B02G205000 | 4 |
|  | TaRbohF3 | TraesCS6D02G164500 | 4 |
|  | TaRbohG1 | TraesCS5A02G211800 | 4 |
|  | TaRbohG2 | TraesCS5B02G212100 | 4 |
|  | TaRbohG3 | TraesCS5D02G222100 | 4 |
|  | TaRbohH1 | TraesCS5A02G093600 | 4 |
|  | TaRbohH2 | TraesCS5B02G099700 | 4 |
|  | TaRbohH3 | TraesCS5D02G105900 | 4 |
|  | TaRbohI1 | TraesCS3A02G182900 | 4 |
|  | TaRbohI2 | TraesCS3B02G212900 | 4 |
|  | TaRbohI3 | TraesCS3D02G187300 | 4 |
|  | TaRbohJ2 | TraesCS1B02G295200 | 3 |
|  | TaRbohJ3 | TraesCS1D02G284900 | 3 |
|  | TaRbohK2 | TraesCS1B02G295300 | 3 |
|  | TaRbohK3 | TraesCS1D02G285000 | 3 |
|  | TaRbohL1 | TraesCS1A02G285700 | 4 |
|  | TaRbohL3 | TraesCS1D02G284800 | 4 |
|  | TaRbohM1 | TraesCS5A02G301700 | 4 |
|  | TaRbohM2 | TraesCS5B02G299000 | 4 |
|  | TaRbohM3 | TraesCS5D02G306400 | 4 |
|  | TaRbohN1 | TraesCS5A02G499900 | 4 |
|  | TaRbohN2 | TraesCS4B02G327800 | 4 |
|  | TaRbohN3 | TraesCS4D02G324800 | 4 |
| 0 singleton；1 dispersed；2 proximal；3 tandem；4 WGD/segmental | | | |

**Table S3** Duplicated Rboh gene pairs Intra-species.

| Species | Seq1 | Seq2 | Duplicate type |
| --- | --- | --- | --- |
| *Zea mays* | ZmRbohD | ZmRbohK | 4 |
|  | ZmRbohE | ZmRbohN | 4 |
|  | ZmRbohI | ZmRbohM | 4 |
| *Oryza sativa* | OsRbohA | OsRbohC | 4 |
|  | OsRbohD | OsRbohE | 4 |
|  | OsRbohF | OsRbohG | 4 |
| *Sorghum bicolor* | SbRbohG | SbRbohE | 4 |
|  | SbRbohC | SbRbohB | 4 |
| *Brachypodium distachyon* | BdRbohC | BdRbohB | 4 |
|  | BdRbohF | BdRbohG | 4 |
| *Arabidopsis thaliana* | AtRbohC | AtRbohG | 4 |
|  | AtRbohF | AtRbohI | 4 |
|  | AtRbohH | AtRbohJ | 4 |
| *Setaria italica* | SiRbohB | SiRbohC | 4 |
|  | SiRbohF | SiRbohM | 4 |
| *Hordeum vulgare* | HvRbohD | HvRbohE | 4 |
|  | HvRbohC | HvRbohA | 4 |
| *Triticum aestivum* | TaRbohD1 | TaRbohD2 | 4 |
|  | TaRbohC1 | TaRbohC2 | 4 |
|  | TaRbohL1 | TaRbohL3 | 4 |
|  | TaRbohD1 | TaRbohD3 | 4 |
|  | TaRbohC1 | TaRbohC3 | 4 |
|  | TaRbohC1 | TaRbohA1 | 4 |
|  | TaRbohD1 | TaRbohE1 | 4 |
|  | TaRbohC1 | TaRbohA2 | 4 |
|  | TaRbohD1 | TaRbohE2 | 4 |
|  | TaRbohC1 | TaRbohA3 | 4 |
|  | TaRbohD1 | TaRbohE3 | 4 |
|  | TaRbohD2 | TaRbohD3 | 4 |
|  | TaRbohC2 | TaRbohC3 | 4 |
|  | TaRbohC2 | TaRbohA1 | 4 |
|  | TaRbohD2 | TaRbohE1 | 4 |
|  | TaRbohC2 | TaRbohA2 | 4 |
|  | TaRbohD2 | TaRbohE2 | 4 |
|  | TaRbohC2 | TaRbohA3 | 4 |
|  | TaRbohC3 | TaRbohA1 | 4 |
|  | TaRbohC3 | TaRbohA2 | 4 |
|  | TaRbohD3 | TaRbohE2 | 4 |
|  | TaRbohC3 | TaRbohA3 | 4 |
|  | TaRbohD3 | TaRbohE3 | 4 |
|  | TaRbohI1 | TaRbohI2 | 4 |
|  | TaRbohA1 | TaRbohA2 | 4 |
|  | TaRbohA1 | TaRbohA3 | 4 |
|  | TaRbohI1 | TaRbohI3 | 4 |
|  | TaRbohA2 | TaRbohA3 | 4 |
|  | TaRbohE2 | TaRbohE3 | 4 |
|  | TaRbohI2 | TaRbohI3 | 4 |
|  | TaRbohN2 | TaRbohN3 | 4 |
|  | TaRbohB2 | TaRbohB3 | 4 |
|  | TaRbohN2 | TaRbohN1 | 4 |
|  | TaRbohB2 | TaRbohB1 | 4 |
|  | TaRbohN3 | TaRbohN1 | 4 |
|  | TaRbohB3 | TaRbohB1 | 4 |
|  | TaRbohH1 | TaRbohH2 | 4 |
|  | TaRbohG1 | TaRbohG2 | 4 |
|  | TaRbohM1 | TaRbohM2 | 4 |
|  | TaRbohH1 | TaRbohH3 | 4 |
|  | TaRbohG1 | TaRbohG3 | 4 |
|  | TaRbohM1 | TaRbohM3 | 4 |
|  | TaRbohG2 | TaRbohG3 | 4 |
|  | TaRbohM2 | TaRbohM3 | 4 |
|  | TaRbohH2 | TaRbohH3 | 4 |
|  | TaRbohF1 | TaRbohF2 | 4 |
|  | TaRbohF1 | TaRbohF3 | 4 |
|  | TaRbohF2 | TaRbohF3 | 4 |
| 0 singleton；1 dispersed；2 proximal；3 tandem；4 WGD/segmental | | | |

**Table S4** Duplicated Rboh gene pairs among seven gramineous plants.

| *Zea mays* | *Sorghum bicolor* | *Oryza sativa* | *Brachypodium distachyon* | *Setaria italica* | *Hordeum vulgare* | *Triticum aestivum* |
| --- | --- | --- | --- | --- | --- | --- |
| ZmRbohA | SbRbohA | OsRbohD | BdRbohA | SiRbohA | HvRbohD | TaRbohD1 |
| ZmRbohA |  | OsRbohE | BdRbohE |  | HvRbohE | TaRbohD3 |
| ZmRbohA |  |  |  |  |  | TaRbohE2 |
| ZmRbohA |  |  |  |  |  | TaRbohE3 |
| ZmRbohB | SbRbohB | OsRbohA | BdRbohB | SiRbohC | HvRbohC | TaRbohC2 |
| ZmRbohB | SbRbohC | OsRbohC | BdRbohC | SiRbohB | HvRbohA | TaRbohC3 |
| ZmRbohB |  |  |  |  |  | TaRbohC1 |
| ZmRbohB |  |  |  |  |  | TaRbohA1 |
| ZmRbohB |  |  |  |  |  | TaRbohA2 |
| ZmRbohB |  |  |  |  |  | TaRbohA3 |
| ZmRbohC | SbRbohB | OsRbohA | BdRbohB | SiRbohC | HvRbohC | TaRbohC1 |
| ZmRbohC | SbRbohC | OsRbohC | BdRbohC | SiRbohB | HvRbohA | TaRbohC2 |
| ZmRbohC |  |  |  |  |  | TaRbohC3 |
| ZmRbohC |  |  |  |  |  | TaRbohA1 |
| ZmRbohC |  |  |  |  |  | TaRbohA2 |
| ZmRbohC |  |  |  |  |  | TaRbohA3 |
| ZmRbohD | SbRbohD | OsRbohI | BdRbohD | SiRbohD |  |  |
| ZmRbohE | SbRbohE | OsRbohF | BdRbohF | SiRbohM |  | TaRbohG2 |
| ZmRbohE | SbRbohG | OsRbohG | BdRbohG | SiRbohF | HvRbohG | TaRbohG3 |
| ZmRbohF | SbRbohF | OsRbohH | BdRbohH | SiRbohE | HvRbohH | TaRbohH1 |
| ZmRbohF |  |  |  |  |  | TaRbohH2 |
| ZmRbohF |  |  |  |  |  | TaRbohH3 |
| ZmRbohI | SbRbohI | OsRbohB | BdRbohI | SiRbohI |  |  |
| ZmRbohJ | SbRbohD | OsRbohI | BdRbohD | SiRbohD |  |  |
| ZmRbohL |  |  |  | SiRbohH |  |  |
| ZmRbohM | SbRbohI | OsRbohB | BdRbohI | SiRbohI |  |  |
| ZmRbohN | SbRbohG | OsRbohF | BdRbohF | SiRbohM | HvRbohG | TaRbohG1 |
| ZmRbohN | SbRbohE | OsRbohG | BdRbohG | SiRbohF |  | TaRbohG2 |
| ZmRbohN |  |  |  |  |  | TaRbohG3 |

**Table S5** Estimates of the dates for the large-scale duplication events between Rboh genes in maize and other six plants

| Seq1 | Seq2 | *Ka* | *Ks* | *Ka/Ks* | Time(Mya) |
| --- | --- | --- | --- | --- | --- |
| ZmRbohA | OsRbohE | 0.0486 | 0.8179 | 0.0595 | 44.9396 |
| ZmRbohA | OsRbohD | 0.1180 | 1.1434 | 0.1032 | 62.8242 |
| ZmRbohA | BdRbohA | 0.1186 | 0.9619 | 0.1233 | 52.8516 |
| ZmRbohA | BdRbohE | 0.0660 | 0.7424 | 0.0889 | 40.7912 |
| ZmRbohA | SbRbohA | 0.0143 | 0.1988 | 0.0721 | 10.9231 |
| ZmRbohA | SiRbohA | 0.0301 | 0.3582 | 0.0840 | 19.6797 |
| ZmRbohA | HvRbohD | 0.1681 | 0.7613 | 0.2209 | 41.8297 |
| ZmRbohA | HvRbohE | 0.0669 | 0.4934 | 0.1356 | 27.1099 |
| ZmRbohA | TaRbohD1 | 0.1663 | 0.7754 | 0.2144 | 42.6037 |
| ZmRbohA | TaRbohD3 | 0.1666 | 0.7858 | 0.2121 | 43.1779 |
| ZmRbohA | TaRbohE2 | 0.0642 | 0.4944 | 0.1297 | 27.1670 |
| ZmRbohA | TaRbohE3 | 0.0685 | 0.4848 | 0.1414 | 26.6353 |
| ZmRbohB | OsRbohA | 0.0280 | 0.5394 | 0.0519 | 29.6374 |
| ZmRbohB | OsRbohC | 0.0715 | 0.7693 | 0.0929 | 42.2692 |
| ZmRbohB | BdRbohB | 0.0489 | 0.5345 | 0.0914 | 29.3681 |
| ZmRbohB | BdRbohC | 0.0741 | 0.7792 | 0.0951 | 42.8132 |
| ZmRbohB | SbRbohB | 0.0069 | 0.0770 | 0.0892 | 4.2308 |
| ZmRbohB | SbRbohC | 0.0786 | 0.8427 | 0.0933 | 46.3022 |
| ZmRbohB | SiRbohC | 0.0831 | 0.6991 | 0.1188 | 38.4118 |
| ZmRbohB | SiRbohB | 0.0143 | 0.2533 | 0.0565 | 13.9184 |
| ZmRbohB | HvRbohC | 0.0921 | 0.7912 | 0.1164 | 43.4702 |
| ZmRbohB | HvRbohA | 0.0653 | 0.6452 | 0.1012 | 35.4512 |
| ZmRbohB | TaRbohC2 | 0.0940 | 0.8003 | 0.1175 | 43.9706 |
| ZmRbohB | TaRbohC3 | 0.0912 | 0.7740 | 0.1178 | 42.5281 |
| ZmRbohB | TaRbohC1 | 0.0987 | 0.7587 | 0.1301 | 41.6856 |
| ZmRbohB | TaRbohA1 | 0.0652 | 0.5845 | 0.1115 | 32.1168 |
| ZmRbohB | TaRbohA2 | 0.0620 | 0.5966 | 0.1040 | 32.7806 |
| ZmRbohB | TaRbohA3 | 0.0641 | 0.5777 | 0.1110 | 31.7429 |
| ZmRbohC | OsRbohA | 0.0416 | 0.7228 | 0.0576 | 39.7143 |
| ZmRbohC | OsRbohC | 0.0490 | 0.5032 | 0.0973 | 27.6484 |
| ZmRbohC | BdRbohC | 0.0458 | 0.5075 | 0.0903 | 27.8846 |
| ZmRbohC | BdRbohB | 0.0806 | 0.7602 | 0.1061 | 41.7692 |
| ZmRbohC | SbRbohB | 0.0767 | 0.8235 | 0.0931 | 45.2473 |
| ZmRbohC | SbRbohC | 0.0084 | 0.1297 | 0.0648 | 7.1264 |
| ZmRbohC | SiRbohC | 0.0157 | 0.2832 | 0.0555 | 15.5609 |
| ZmRbohC | SiRbohB | 0.0787 | 0.8048 | 0.0978 | 44.2183 |
| ZmRbohC | HvRbohC | 0.0562 | 0.5877 | 0.0956 | 32.2932 |
| ZmRbohC | HvRbohA | 0.0881 | 0.8493 | 0.1037 | 46.6661 |
| ZmRbohC | TaRbohC1 | 0.0652 | 0.5836 | 0.1117 | 32.0666 |
| ZmRbohC | TaRbohC2 | 0.0606 | 0.5846 | 0.1037 | 32.1228 |
| ZmRbohC | TaRbohC3 | 0.0603 | 0.5948 | 0.1014 | 32.6836 |
| ZmRbohC | TaRbohA1 | 0.0871 | 0.7913 | 0.1101 | 43.4788 |
| ZmRbohC | TaRbohA2 | 0.0928 | 0.8205 | 0.1131 | 45.0813 |
| ZmRbohC | TaRbohA3 | 0.0953 | 0.7867 | 0.1212 | 43.2258 |
| ZmRbohD | OsRbohI | 0.0625 | 1.9248 | 0.0325 | 105.7582 |
| ZmRbohD | BdRbohD | 0.0760 | 1.8252 | 0.0416 | 100.2857 |
| ZmRbohD | SbRbohD | 0.0361 | 1.0417 | 0.0347 | 57.2363 |
| ZmRbohD | ZmRbohK | 0.1247 | 0.8827 | 0.1413 | 48.5000 |
| ZmRbohD | SiRbohD | 0.0517 | 0.3139 | 0.1647 | 17.2456 |
| ZmRbohE | OsRbohF | 0.1245 | 1.1056 | 0.1126 | 60.7473 |
| ZmRbohE | OsRbohG | 0.1062 | 1.5542 | 0.0683 | 85.3956 |
| ZmRbohE | BdRbohF | 0.1057 | 1.0402 | 0.1017 | 57.1538 |
| ZmRbohE | BdRbohG | 0.1145 | 1.4776 | 0.0775 | 81.1868 |
| ZmRbohE | SbRbohG | 0.1141 | 1.7292 | 0.0660 | 95.0110 |
| ZmRbohE | SbRbohE | 0.0410 | 0.4224 | 0.0971 | 23.2088 |
| ZmRbohE | ZmRbohN | 0.1111 | 1.7378 | 0.0639 | 95.4835 |
| ZmRbohE | SiRbohM | 0.1259 | 0.6929 | 0.1817 | 38.0737 |
| ZmRbohE | SiRbohF | 0.0508 | 0.3509 | 0.1449 | 19.2796 |
| ZmRbohE | HvRbohG | 0.1381 | 0.6896 | 0.2003 | 37.8916 |
| ZmRbohE | TaRbohG2 | 0.1359 | 0.6687 | 0.2033 | 36.7424 |
| ZmRbohE | TaRbohG3 | 0.1363 | 0.6481 | 0.2103 | 35.6117 |
| ZmRbohF | OsRbohH | 0.1073 | 1.3875 | 0.0774 | 76.2363 |
| ZmRbohF | BdRbohH | 0.1025 | 1.9545 | 0.0524 | 107.3901 |
| ZmRbohF | SbRbohF | 0.0359 | 0.4878 | 0.0736 | 26.8022 |
| ZmRbohF | SiRbohE | 0.0571 | 0.2981 | 0.1915 | 16.3771 |
| ZmRbohF | HvRbohH | 0.1063 | 0.3816 | 0.2786 | 20.9658 |
| ZmRbohF | TaRbohH1 | 0.1060 | 0.3979 | 0.2664 | 21.8641 |
| ZmRbohF | TaRbohH2 | 0.1092 | 0.3857 | 0.2832 | 21.1901 |
| ZmRbohF | TaRbohH3 | 0.1022 | 0.3869 | 0.2642 | 21.2568 |
| ZmRbohI | OsRbohB | 0.0734 | 0.6934 | 0.1058 | 38.0989 |
| ZmRbohI | BdRbohI | 0.0656 | 0.5668 | 0.1158 | 31.1429 |
| ZmRbohI | SbRbohI | 0.0266 | 0.1351 | 0.1965 | 7.4231 |
| ZmRbohI | ZmRbohM | 0.0314 | 0.1565 | 0.2009 | 8.5989 |
| ZmRbohI | SiRbohI | 0.0438 | 0.3989 | 0.1099 | 21.9155 |
| ZmRbohJ | OsRbohI | 0.1193 | 2.6518 | 0.0450 | 145.7033 |
| ZmRbohJ | BdRbohD | 0.1312 | 2.3222 | 0.0565 | 127.5934 |
| ZmRbohJ | SbRbohD | 0.0929 | 1.1176 | 0.0831 | 61.4066 |
| ZmRbohJ | SiRbohD | 0.1188 | 0.3334 | 0.3565 | 18.3171 |
| ZmRbohL | SiRbohH | 0.0416 | 0.5010 | 0.0830 | 27.5273 |
| ZmRbohM | OsRbohB | 0.0668 | 0.6435 | 0.1039 | 35.3571 |
| ZmRbohM | BdRbohI | 0.0608 | 0.5695 | 0.1068 | 31.2912 |
| ZmRbohM | SbRbohI | 0.0215 | 0.1358 | 0.1583 | 7.4615 |
| ZmRbohM | SiRbohI | 0.0320 | 0.3798 | 0.0843 | 20.8670 |
| ZmRbohN | OsRbohF | 0.1468 | 1.2388 | 0.1185 | 68.0659 |
| ZmRbohN | OsRbohG | 0.0540 | 0.7585 | 0.0711 | 41.6758 |
| ZmRbohN | BdRbohF | 0.1264 | 1.1768 | 0.1074 | 64.6593 |
| ZmRbohN | BdRbohG | 0.0489 | 0.6846 | 0.0714 | 37.6154 |
| ZmRbohN | SbRbohG | 0.0160 | 0.2268 | 0.0707 | 12.4615 |
| ZmRbohN | SbRbohE | 0.1204 | 1.3132 | 0.0917 | 72.1538 |
| ZmRbohN | SiRbohM | 0.0195 | 0.2531 | 0.0770 | 13.9081 |
| ZmRbohN | SiRbohF | 0.1218 | 0.6256 | 0.1948 | 34.3713 |
| ZmRbohN | HvRbohG | 0.0656 | 0.4344 | 0.1510 | 23.8660 |
| ZmRbohN | TaRbohG1 | 0.0719 | 0.3941 | 0.1824 | 21.6531 |
| ZmRbohN | TaRbohG2 | 0.0600 | 0.3949 | 0.1520 | 21.6986 |
| ZmRbohN | TaRbohG3 | 0.0622 | 0.4046 | 0.1536 | 22.2335 |

**Table S6** Primers used in this study

| Primer Name | Sequence (5'-3') |
| --- | --- |
| qRT-*ACT*-F | GATTCCTGGGATTGCCGAT |
| qRT-*ACT*-R | TCTGCTGCTGAAAAGTGCTGAG |
| qRT-*TUB*-F | CACTGATGTTGCTGTCCTGC |
| qRT-*TUB*-R | CGCTGTTGGTGATTTCGG |
| qRT-*ZmPT6*-F | GATCCAGCTCATCGGTTTCT |
| qRT-*ZmPT6*-R | GAGCGTGGTGTGTTTGTTCT |
| qRT-*GinEF*-F | GCTATTTTGATCATTGCCGCC |
| qRT-*GinEF*-R | TCATTAAAACGTTCTTCCGACC |
| qRT-*ZmRbohA*-F | CCAAACTGGAGGAAGGTATTCT |
| qRT-*ZmRbohA*-R | ATCCTTCAGTTGTTTTGTGAGC |
| qRT-*ZmRbohB*-F | CTTGGAAGTTCATGCAGTATCG |
| qRT-*ZmRbohB*-R | TTCCCTGCATGGAGGATTATAC |
| qRT-*ZmRbohC*-F | ATCGAAGGTTCAAGAGCACATA |
| qRT-*ZmRbohC*-R | TATGTGTCCTACATCACGCTAC |
| qRT-*ZmRbohD*-F | GAACAAGCTTTCGAAGATCACG |
| qRT-*ZmRbohD*-R | CAGGTTGTACAGCTCGATGTAG |
| qRT-*ZmRbohE*-F | CAGGACTTCAGGAACTACGAC |
| qRT-*ZmRbohE*-R | TTGATGTTGTTGAGGAGGTCTC |
| qRT-*ZmRbohF*-F | GGATGGTCGGATCCGAGGAGTTTG |
| qRT-*ZmRbohF*-R | CTCGATGTAGCCAAGGCTGTCAGG |
| qRT-*ZmRbohG*-F | GACTGCAGACTTTCTTCCACAT |
| qRT-*ZmRbohG*-R | TGATCTTCGACAGCTTGTTCTC |
| qRT-*ZmRbohH*-F | AGTCACGAATTGGGACGTGG |
| qRT-*ZmRbohH*-R | AAACGCTTAGAACCCTCCCC |
| qRT-*ZmRbohI*-F | TTTTACGGCACAAATGAGTCAG |
| qRT-*ZmRbohI*-R | TTTTGAGCAAACTTACGGAAGG |
| qRT-*ZmRbohJ*-F | GCATTGGGATGAATGAGGCG |
| qRT-*ZmRbohJ*-R | TGATGATCTGCATGCGTTTTT |
| qRT-*ZmRbohK*-F | CCATGATCCAGTCGTTCAACTA |
| qRT-*ZmRbohK*-R | TGAGAATTCCTGCGTGAGTTC |
| qRT-*ZmRbohL*-F | CATCATAAAGGCGGCAATCTAC |
| qRT-*ZmRbohL*-R | CCAATCACCTAACGTACGGATA |
| qRT-*ZmRbohM*-F | AGGTCCATTTTACCACTACTGG |
| qRT-*ZmRbohM*-R | CCATGATGTGAAATACAGCTCG |
| qRT-*ZmRbohN*-F | AGAAGCTTGCCAAAATTACTCG |
| qRT-*ZmRbohN*-R | CGTAGAATGCTTATGAACGGTG |
| p*ZmRbohF*-F | GCAGGCATGCAAGCTTGAGATGAGTGTTTCTGTGCGAG |
| p*ZmRbohF*-R | CTCAGATCTACCATGGTGCGTGAGGCACGCTAGTATGA |
| *EMS-ZmRbohF-F* | *GCCTGGTTGAGGAACTGGA* |
| *EMS-ZmRbohF-R* | *GTCACAGCACACAGACAGCATT* |

**Table S7** B&D culture medium.

| Reagent | Concentration |
| --- | --- |
| CaCl_2_•2H_2_O | 1.0M |
| K_2_HPO_4_ | 0.5M |
| Fe-Citrate | 0.01M |
| MgSO_4_•7H_2_O | 0.25M |
| K_2_SO_4_ | 1.5M |
| KNO_3_ | 1M |
| MnSO_4_•H_2_O | 10^-3^M |
| H_3_BO_3_ | 2x10^-3^M |
| CuSO_4_•5H_2_O | 2x10^-4^M |
| CoSO_4_•7H_2_O | 10^-4^M |
| ZnSO_4_•7H_2_O | 10^-4^M |
| NaMoO_4_•2H_2_O | 5x10^-4^M |
